# Cell modeling using frequency modulation

**DOI:** 10.1371/journal.pone.0315003

**Published:** 2024-12-06

**Authors:** Jerry Jacob, Nitish Patel, Sucheta Sehgal

**Affiliations:** Department of Electrical, Computer and Software Engineering, The University of Auckland, Auckland, New Zealand; Guangdong University of Petrochemical Technology, CHINA

## Abstract

Computational models of the cell can be used to study the impact of drugs and assess pathological risks. Typically, these models are computationally demanding or challenging to implement in dedicated hardware for real-time emulation. A new Frequency Modulation (FM) model is proposed to address these limitations. This model utilizes a single sine generator with constant amplitude, while phase and frequency are modulated to emulate an action potential (AP). The crucial element of this model is the identification of the modulating signal. Focusing on FPGA implementation, we have employed a piecewise linear polynomial with a fixed number of breakpoints to serve as the modulating signal. The adaptability of this signal permits the emulation of dynamic properties and the coupling of cells. Additionally, we have introduced a state controller that handles both of these requirements. The building blocks of the FM model have direct integer equivalents, making them suitable for implementation on digital platforms like Field Programmable Gate Arrays (FPGA). We have demonstrated wavefront propagation in 1-D and 2-D models of tissue. We have used various parameters to quantify the wavefront propagation in 2-D tissues and emulated specific cellular dysfunctions. The FM model can replicate any detailed cell model and emulate its corresponding tissue model. This model is at its preliminary stage. The FPGA implementation of this model is a work in progress. Overall, the results demonstrate that the FM model has the potential for real-time cell and tissue emulation on an FPGA.

## Introduction

### Mathematical models of biological cells

Mathematical models incorporating electrical functions have been pivotal in comprehending cellular mechanisms. The Hodgkin-Huxley (HH) model [1] was a pioneering example, accurately describing action potentials (APs) of a squid axon. Afterward, various other models like FitzHugh Nagumo (FHN) [2], Fenton Karma (FK) [3], Luo-Rudy-I (LR-I) [4], Beeler Reuter (BR) [5], Fabbri et al. [6] and O’Hara [7] models, have expanded on the HH model in many spheres. There exist other cardiac cell models based on purkinje fibres [8–11], ventricular nodes [3–5, 7, 12–29], sinoatrial node [6, 30, 31] and atrioventricular nodes [32]. These models have approximately 2–100 variables and many ordinary differential equations (ODEs).

There exists a possibility of creating solvers for ODEs using the Runge-Kutta 4nd order (RK4), Runge Kutta 2nd order (RK2), Forward Euler 1st order (FE1) and others. However, the work done by Bartel and Koch [33] utilizes the FPGA Intel Arria 10 GX 1150 which has a variety of resources like 65 MB internal RAM, 1.15 million logical blocks and 3036 digital signal processing (DSP) blocks. They utilized three solver techniques, i.e., Huen, Euler and strong stability preserving Runge Kutta 2nd order (SSPRK2) methods. They created these solvers using single point, 54-bit fixed point and double point. They found that the solvers take a lot of DSP blocks and Look-up-Tables (LUTs) which are resource heavy. Among the three SSPRK2 takes the highest resources in terms of these. In terms of the implementing the biophysical models on an FPGA, the works by Othman et. al. [34, 35] and Adon et. al. [36] showed us some crucial insights on the computational space. These authors replicated LR-I and FHN cell models on an FPGA by utilizing the MATLAB VHDL coder. To reproduce these models, they had to avoid the overflow conditions due to various functions like log and exponential. To counter these issues, they had to utilize a variety of LUTs for APD replication. The Yanagihara-Noma-Irisawa (YNI) [37] sinoatrial cell model was emulated on Zynq XC7Z010 board by Ghanbarpour et. al. [38]. They compared the results of the detailed model to their converted model where the CORDIC algorithm [39] was used. The authors observed that the detailed model on the FPGA utilizes 61.25% of DSPs (49 out 80) and 12.12% of slice LUTs comprising of LUTs, multiplexers and flip flops (2134 out of 17600) for cell emulation. On the other hand, the converted model does not use DSPs but uses 8.18% of slice LUTs. Yang et. al. [40] found a series of methods for FPGA implementation of an ODE based neuron model using Cyclone IV EP4CE115. They found that these models require 62% (328 out of 532) of embedded multipliers for its implementation. Overall, the components like LUTs, DSPs and multipliers tend to take more computational space on an FPGA while implementing the detailed cell models.

### Reduced generic cell models

To overcome the aforementioned issue, Sehgal et.al. [41, 42] formulated the Resonant Model (RM), which utilizes the Fourier Series (FS) to reconstruct various detailed models like Kurata et al. [43], Fabbri [6], Fenton Karma [3], O’Hara [7], Courtemanche et al. [44] and Dobrzynski et al. [45] models. The optimization of the Fourier Series coefficients was done by using the Levenberg-Marquardt algorithm [46]. The researchers exhibited the model’s ability to precisely emulate AP morphologies by altering the funny current (*I*_*f*_) block in the Fabbri et al. [6] model using linear and mixed fit-type equations. Jacob et al. [47] expanded upon this model by successfully applying the FS-based approach using non-linear regression [48, 49] to reconstruct APDs for the FHN, FK, BR, and LR-I models. They also quantified the resource usage of RM cells, demonstrating their lower resource requirements compared to Direct Digital Synthesis (DDS) methods. They [50] utilized the diffusion equation to simulate a ventricular tissue model using the RM.

Another emerging model that can be used to reconstruct the APDs is the Frequency Modulation Mobius (FMM) model [51–53]. Researchers have developed an R-software-based package for implementing the FMM [54], showcasing its effectiveness in reconstructing various data, including Iqgap gene expression, electrocardiography (ECG) records, and APs of the HH model.

### Advantage of the novel FM model over the simplified versions

The RM and FMM require multiple sinusoids to reconstruct major AP characteristics. The FM model attempts to reduce this aspect of the reconstruction. It simplifies the reconstruction of action potential durations (APDs) using a single sine wave. Hence, it is a mono-component model. It aims to utilize a sine wave with varying frequency but with a constant amplitude and phase. This study explores the efficacy of this model in terms of RMSE and *R*^2^ values for the reconstructions of the APDs of FitzHugh Nagumo (FHN) [2], Fenton Karma (FK), Beeler-Reuter and Fabbri [6] models. This paper also delves into the feasibility of parameterization, as in Sehgal et al. [41] for varying *I*_*f*_ block. We have designed a state controller for the FM model to emulate more dynamic properties to the replicated AP profiles. Utilizing this state controller, we created various propagation wavefronts for 1-D and 2-D tissues using O’Hara [7] and FHN models. The waveform propagation observation from a 2-D FM tissue model is aligned with that of the detailed models. Lastly, we introduced some cellular dysfunctions to the 2-D tissues and observed the alterations in the parameters.

## Theoretical background

### Concept

**Fig 1:**
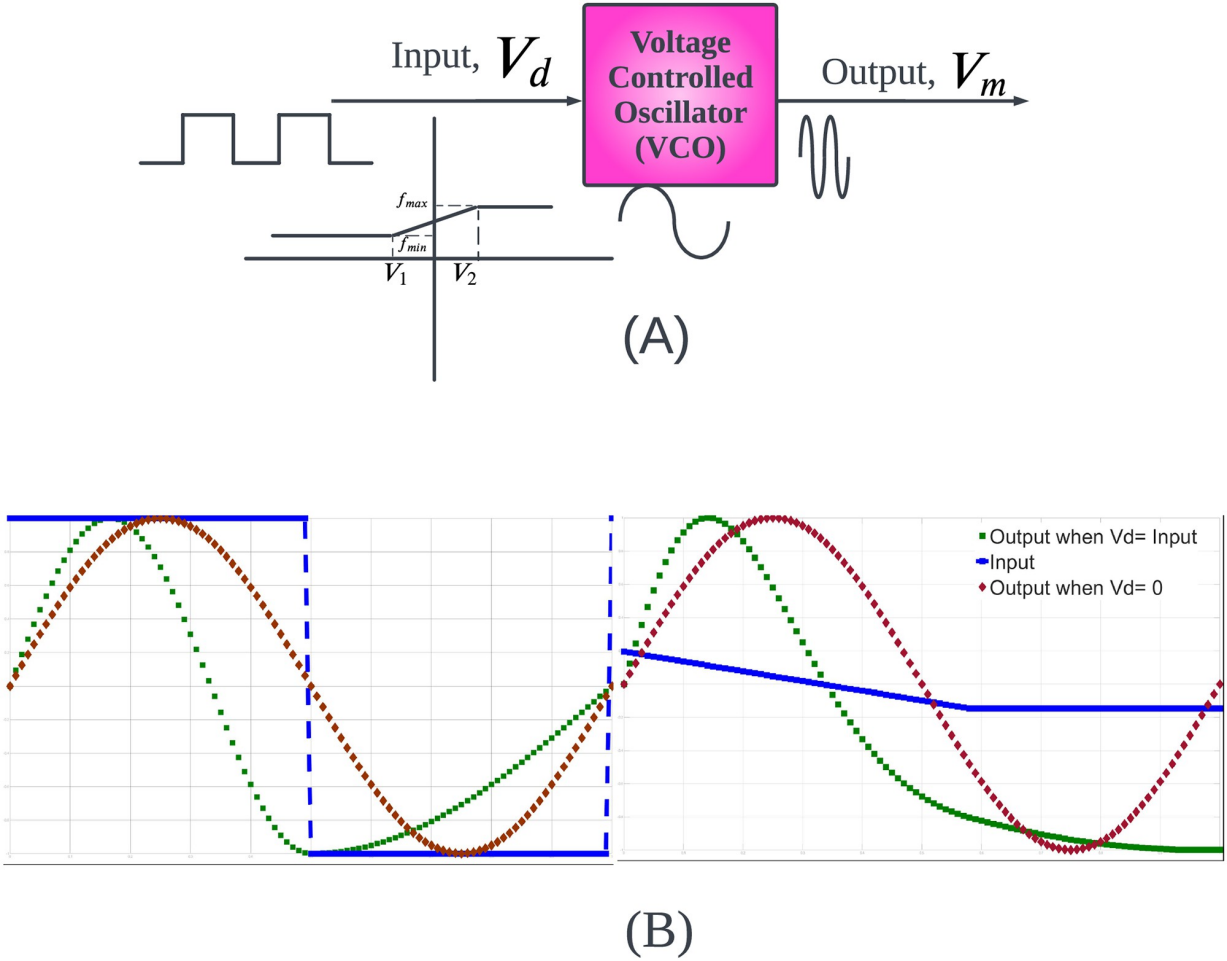
(A) Block diagram eliciting the working of a VCO (B) A sinusoidal wave of two periods altered by firstly a square wave and a combination of ramp and step functions.

A Voltage Controlled Oscillator (VCO) is a device that outputs a repetitive signal with a frequency that depends on an input signal. It is typically used in broadcast communication, modems, PLLs and clock generation. Fig 1(A) shows, conceptually, how we intend to use our model. Fig 1(B) shows three signals: a pure sine wave as a reference, an example input signal and the resulting output of the VCO. The input has 4 segments: a positive step, a negative step, a slow negative ramp and a small negative constant. The frequency of *V*_*m*_ is larger for the positive step and smaller for the negative step. The frequency decreases slowly during the slow negative ramp; hence, the amplitude profile looks non-sinusoidal. As the input reduces and reaches a constant value, the frequency of the modulated sine wave remains constant. By astutely varying the profile like in Fig 1(B), the APDs can be reconstructed.

### Mathematical formulation of the FM model

A sinusoidal waveshape with a variation of phase, *ϕ*_*t*_(*t*) w.r.t a phase, *ϕ* (in radians) can be generated as in Eq 1.

$$\begin{array}{c} V_{m}\left(t\right)=Csin\left(\omega t+\left(\phi +\phi_{t}\left(t\right)\right)\right) \end{array}$$ (1)

where C is the amplitude, *V*_*m*_(*t*) is the sinusoidal signal generated, *ω* is the constant frequency in rad/s, t is the time in seconds (s) where *t* > 0 and *ϕ*_*t*_(*t*) is the varying phase of the sinusoidal waveshape.

*ϕ*_*t*_(*t*) can be obtained from Eq 2.

$$\begin{array}{c} \phi_{t}\left(t\right)={\text{sin}}^{-1}(\frac{V_{m}\left(t\right)}{C})-\omega t-\phi \end{array}$$ (2)

If Eq 1 is represented as the sinusoidal waveshape, which varies in terms of phase, *ϕ*_*t*_(*t*), then the frequency modulating factor, Δ_*w*_(*t*) can vary the frequency as shown below in Eq 3. We can represent *ϕ*_*t*_(*t*) (in radians) in terms of varying frequency (in rad/s) as in Eq 4.

$$\begin{array}{c} V_{m}\left(t\right)=C\;\text{sin}\left(\left(\omega +\Delta_{w}\left(t\right)\right)t+\phi \right) \end{array}$$ (3)

$$\begin{array}{c} \Delta_{w}\left(t\right)=\frac{\phi_{t}\left(t\right)}{t} \end{array}$$ (4)

where *t* > 0 in *s* and Δ_*w*_(*t*) is the frequency modulating factor.

The Δ_*w*_ can be replicated using *n* linear equations as shown in Eq 5. This equation is useful to create the Δ_*w*_ profile on an FPGA.

$$\begin{array}{c} \Delta_{w}\left(t\right)=\{\begin{array}{cc} A_{1}t, & 0<t\leq T_{1}\\ A_{2}t+B_{2}, & T_{1}<t\leq T_{2}\\ A_{3}t+B_{3}, & T_{2}<t\leq T_{2}\\ ...............................\\ ...............................\\ ...............................\\ ...............................\\ A_{n}t+B_{n}, & T_{n-1}<t\leq T_{n} \end{array} \end{array}$$ (5)

where *T*_1_,*T*_2_,….,*T*_*n*_ are the time instants corresponding to the breakpoints and *B*_2_ = *A*_1_*T*_1_, *B*_3_ = *A*_2_*T*_2_ + *B*_2_, ………, *B*_*n*_ = *A*_*n*−1_*T*_*n*−1_ + *B*_*n*−1_.

Also we can represent Eq 3 as in Eq 6.

$$\begin{array}{c} V_{m}\left(t\right)=C\;\text{sin}\left(\left(V_{d}\left(t\right)\right)t+\phi \right) \end{array}$$ (6)

where *V*_*d*_(*t*) = (*ω* + Δ_*w*_(*t*)), *t* is time in *s*, *ϕ* is the constant phase (in radians), and *V*_*m*_(*t*) is the generated sinusoidal function in terms of varying frequency (in rad/s).

### Test case 1

This model relies on an accurate determination of Δ_*w*_. However, it is typically non-linear. Since Δ_*w*_(*t*) is updated at every time sample, this would imply that Δ_*w*_ will be a large 1-D matrix (12000 x 1) with a dimension proportional to the samples in one period. It is not feasible for a digital platform. A tractable solution is a set of linear piecewise equations fitted from the non-linear profile of Δ_*w*_.

**Fig 2:**
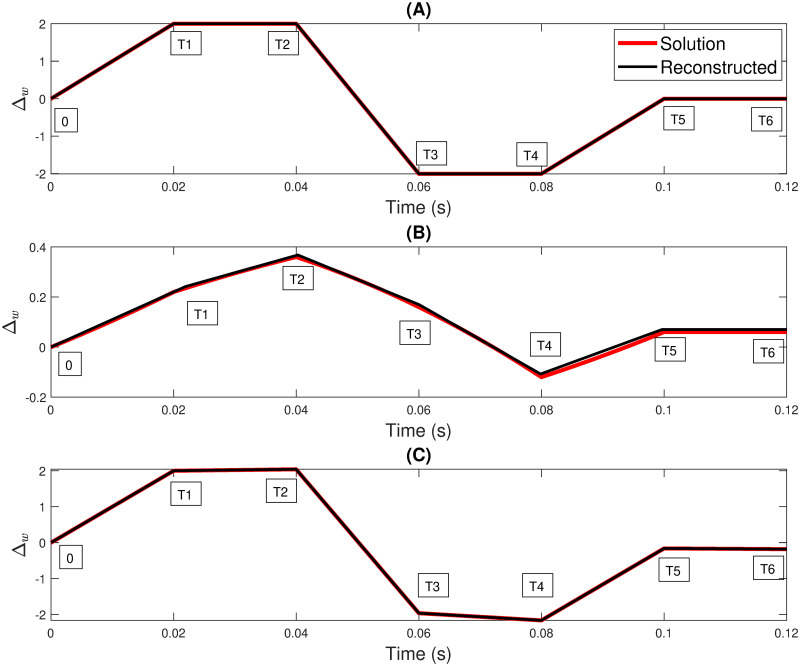
Utilization of three initial test signals (A) Signal 1, (B) Signal 2, and (C) Signal 3 to evaluate the efficacy of the optimization technique of the novel FM model.

This section demonstrates our identification methodology for our objective modulating signal. We intentionally choose three linear test signals (objective modulating signals) of Δ_*w*_(*t*) as shown in Fig 2(A)–2(C) with 12000 samples per period. Six linear equations with 7 breakpoints must be obtained for these test cases. The equation Eq 7 is as follows.

$$\begin{array}{c} \Delta_{w}\left(t\right)=\{\begin{array}{cc} A_{1}t, & 0<t\leq T_{1}\\ A_{2}t+B_{2}, & T_{1}<t\leq T_{2}\\ A_{3}t+B_{3}, & T_{2}<t\leq T_{3}\\ A_{4}t+B_{4}, & T_{3}<t\leq T_{4}\\ A_{5}t+B_{5}, & T_{4}<t\leq T_{5}\\ A_{6}t+B_{6}, & T_{5}<t\leq T_{6} \end{array} \end{array}$$ (7)

where *B*_2_ = *A*_1_*T*_1_, *B*_3_ = *A*_2_*T*_2_ + *B*_2_, *B*_4_ = *A*_3_*T*_3_ + *B*_3_, *B*_5_ = *A*_4_*T*_4_ + *B*_4_ and *B*_6_ = *A*_5_*T*_5_ + *B*_5_.

The Look-Up-Table (LUT) for this is designed as in Table 1. Piecewise linear equations offer the simplest implementation of FPGAs. The optimization of the coefficients of these equations is based on the sum of square error (SSE), the root mean square error (RMSE) and the coefficient of determination (*R*^2^). These are formulated in Eqs 8–10.

$$\begin{array}{c} SSE=\sum_{i=1}^{n}{\left(y_{i}-{\hat{y}}_{i}\right)}^{2} \end{array}$$ (8)

$$\begin{array}{c} RMSE=\sqrt{\frac{1}{n}\sum_{i=1}^{n}{\left(y_{i}-{\hat{y}}_{i}\right)}^{2}} \end{array}$$ (9)

$$\begin{array}{c} R^{2}=1-\frac{\sum_{i=1}^{n}{\left(y_{i}-{\hat{y}}_{i}\right)}^{2}}{\sum_{i=1}^{n}{\left(y_{i}-\bar{y}\right)}^{2}} \end{array}$$ (10)

where *n* represents the total number of samples, *y*_*i*_ denotes the observed values, $\hat{y_{i}}$ represents the predicted values, and $\bar{y}$ represents the mean of the observed values. The SSE values for all three signals (in Table 2) are below 0.9. Signals 1 and 3 portray a significantly reduced SSE of approximately 0.02. Regarding RMSE, Signal 2 is relatively high compared to its trapezoidal counterparts. The *R*^2^ values for all three signals exceed 0.99. These results demonstrate that the reconstructed piecewise linear polynomial generated using this technique can reconstruct the signal with greater precision.

**Table 1:** Coefficients corresponding to Signal 1, 2 and 3.

Segment	Coefficients
0 < t ≤ *T*_1_	*A*_1_
*T*_1_ < t ≤ *T*_2_	*A*_2_
*T*_2_ < t ≤ *T*_3_	*A*_3_
*T*_3_ < t ≤ *T*_4_	*A*_4_
*T*_4_ < t ≤ *T*_5_	*A*_5_
*T*_5_ < t ≤ *T*_6_	*A*_6_

**Table 2:** Verifying the utility of the linear fitting method for Signal 1, 2 and 3.

Signal	SSE	RMSE	*R*^2^ value
Signal 1	0.0241	0.0014	1.0000
Signal 2	0.9602	0.0089	0.9957
Signal 3	0.0201	0.0012	1.0000

For realistic APDs, as discussed in later sections, Δ_*w*_ is non-linear and will require additional considerations. In addition, this method also facilitates the incorporation of the dynamic properties of APDs.

### Test case 2

Here, we consider non-linear modulating signals. We have specifically chosen sinusoids as they offer an additional metric total harmonic distortion (THD) along with RMSE to comprehend the efficiency of fitted waveshape to the sine waves. THD is formulated in Eq 11.

$$\begin{array}{c} THD=\sqrt{\frac{\sum_{k=2}^{N}V_{k}^{2}}{V_{1}^{2}}} \end{array}$$ (11)

where *V*_*k*_ represents the harmonic components from k = 2,…..,N and *V*_1_ represents the fundamental component of the signal.

**Fig 3:**
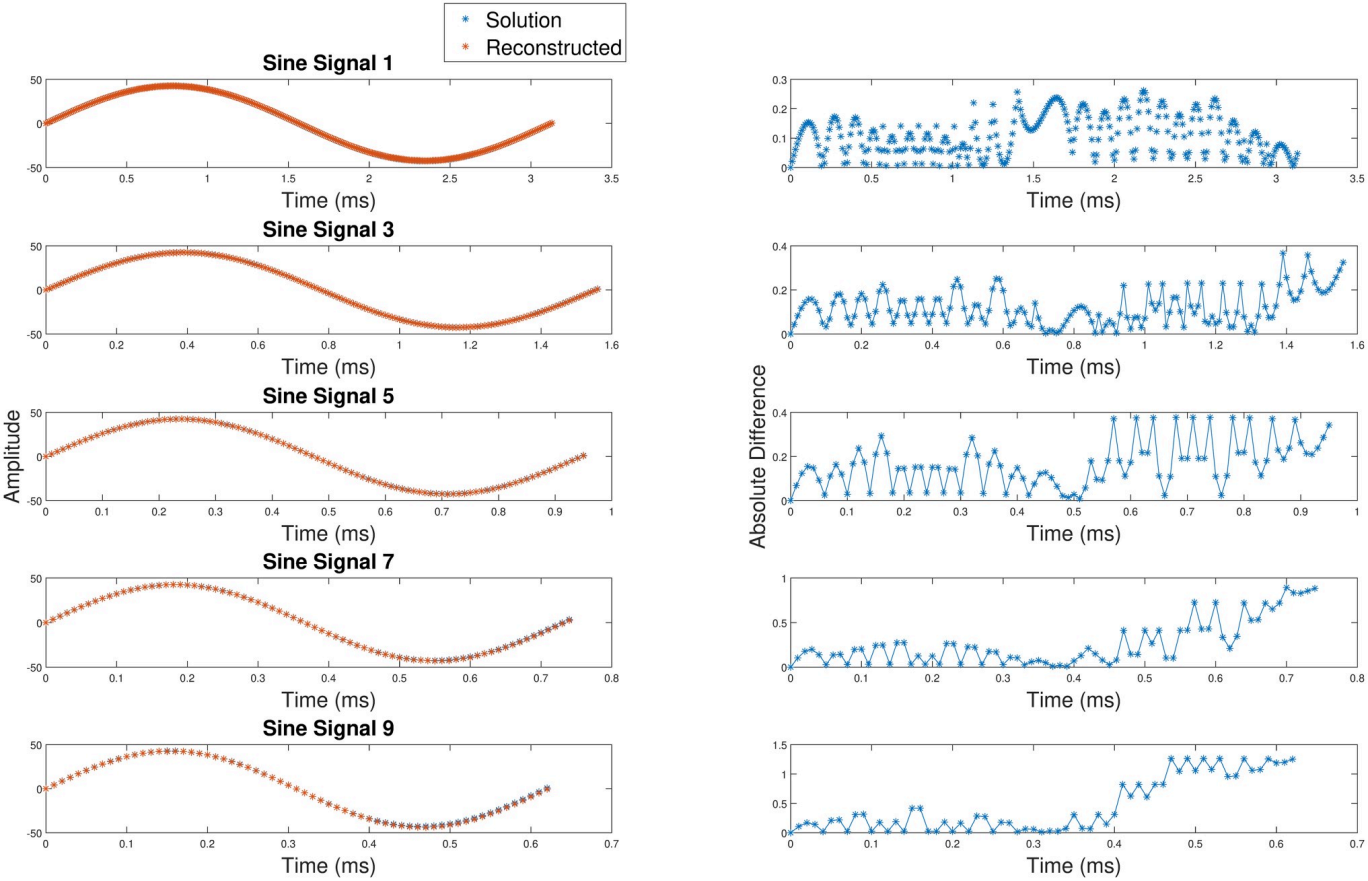
The illustration of sine signals 1, 3, 5, 7 and 9 along with their corresponding point to point absolute difference.

Table 3 considers nine Δ_*w*_(*t*) profiles (refer Fig 3), sinusoids with varied frequencies and amplitudes. Twenty-three linear piecewise segments (24 breakpoints) were considered for all sine waves. The formulation of the linear piecewise segment are as mentioned in Eq 5. For realistic APDs, we require 15–30 linear segments, so we chose 23 segments for this test case.

**Table 3:** The parameters of the tested sine waves in test case 2.

Sine Signal	Frequency (rad/s)	Amplitude (mV)	Total Harmonic Distortion (THD)	RMS Error (RMSE)
Sine Signal 1	2.0102	42.5175	0.0091	0.1286
Sine Signal 2	2.2336	42.5175	0.0108	0.1059
Sine Signal 3	4.0439	42.5271	0.0179	0.1425
Sine Signal 4	5.1015	42.5271	0.0217	0.2496
Sine Signal 5	6.6406	42.5315	0.0258	0.1923
Sine Signal 6	7.7599	42.6564	0.0210	0.1833
Sine Signal 7	8.5969	42.6978	0.0433	0.3630
Sine Signal 8	9.2352	42.7750	0.0494	0.4390
Sine Signal 9	10.1587	42.7970	0.0273	0.6461

The THD remains consistently within the range of approximately 0.01 to 0.05 despite variations in amplitudes and frequencies in the sine waves. The RMSE varies from 0.1 to 0.6 in all nine cases. These metrics affirm that the proposed technique can effectively reconstruct a sine wave signal.

Fig 3 shows the illustrations of the sine signals 1, 3, 5, 7 and 9 along with their corresponding point to point absolute difference. We can observe that the point to point difference in these vary from 0 mV to 1.5 mV. These results show that the piecewise linear fitting technique works for non-linear modulating signals like the sine wave.

### Comparison of the FM model to the state of the art cell models

Various physiological cell models [2–4, 6] comprises of different Ordinary Differential Equations (ODEs). The models include variables which facilitate in varying the APDs. For example, in Fabbri et al. [6] the APD of this model can be varied using the funny current block, *I*_*f*_. Some cell models target single cell type but others can emulate multiple cell types. For each model, the number of ODEs and parameters varies significantly from few to many. This may cause some issues when these are implemented on an FPGA. Fundamentally models [2–4, 6] of these type include non-linear operators which are not easy to parallelize for large tissue models. In some models, this correspondence is difficult to obtain.

On the other hand, the FM model can effectively replicate the APDs of these cell models by using a sinusoidal generator. The results of this paper shows that the FM model tends to replicate the major AP characteristics of any cell effectively. This model also promises to be more friendly while it is implemented on a digital platform. However, further experimentation on whether this model can replicate the APD when the variables in the state of the art cell models are altered is still at its genesis (refer Table 4).

**Table 4:** Comparison of the state of the art models with the FM model.

Properties	State of the art	FM model
APD generation	Through ODEs	Sinusoidal generators
Mathematical complexity	Complex	Simple
Expertise	High	Relatively low
Ease of comprehension	Medium to High	Low
Computational cost of an FPGA	High	Still at its genesis

## Methods

This section initially deals with the methodology for waveshape generation using the FM model. These include acquiring and normalizing various waveshapes to generating the Δ_*w*_ waveshapes to replicate the appropriate APD. These steps will form the foundation for including various dynamic properties like parameterization of the SAN cell model [6], cell-coupling for creating 1-D and 2-D tissues and also to create a state controller which facilitates the replicated cells to be converted into a 1-D or a 2-D tissue. Lastly, we will look at the general overview of the methods involved along with some future works.

### Waveshape generation

**Fig 4:**
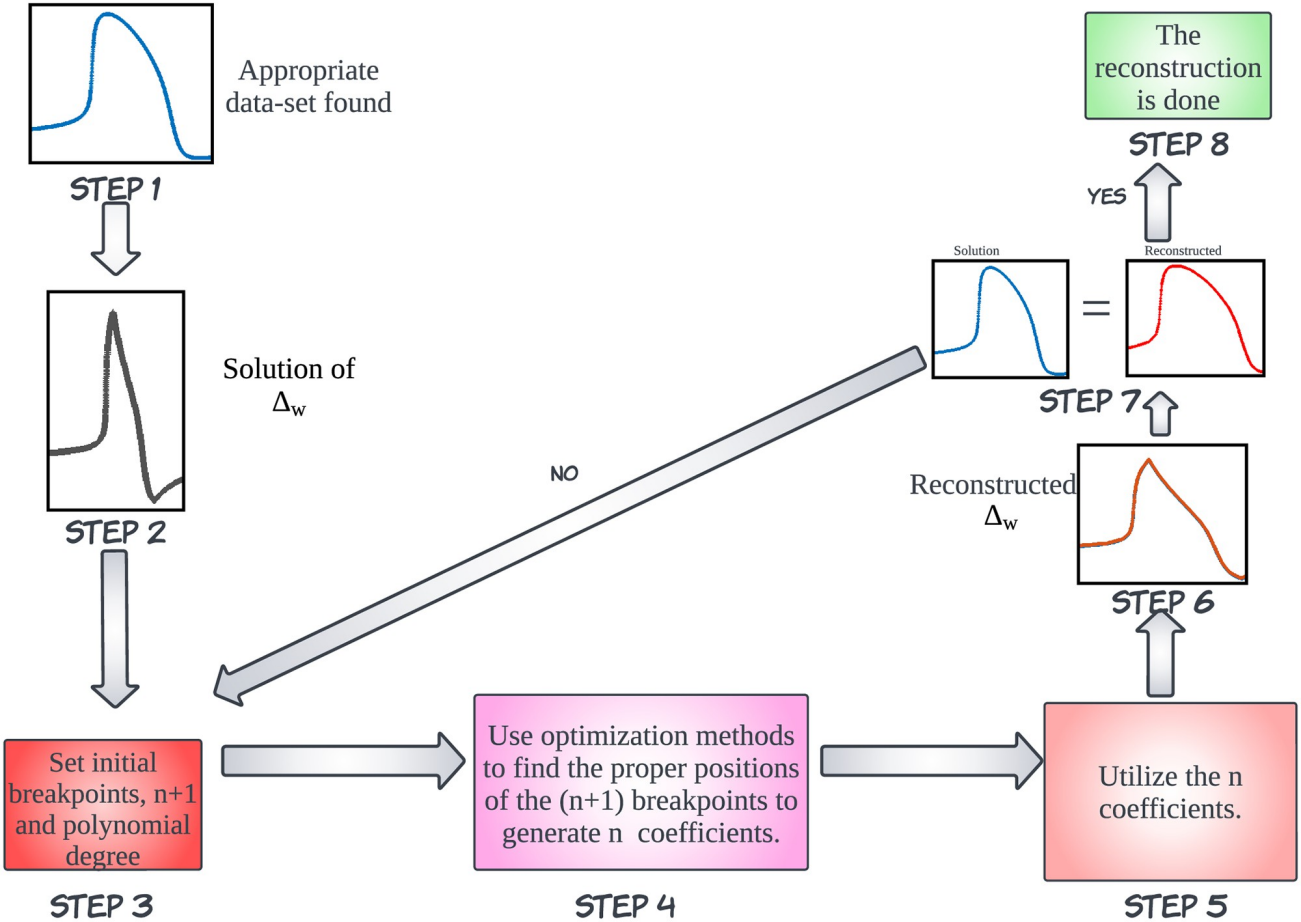
Methodology for waveshape generation using the FM model.

The methodology for the novel FM model is portrayed through a set of steps, as showcased in Fig 4:

Step 1 Acquiring the AP waveshape of the detailed model like in Sehgal et. al. [41, 42]. Normalizing the APD (time series of length K) to a custom numeric range.
Step 2 Generating a Δ_*w*_ waveshape from the acquired dataset as shown in Eq 4.
Step 3 Initializing the number of breakpoints (n+1) for n segments with a chosen polynomial degree.
Step 4 Utilizing an optimization method [55–58] for linear piecewise fitting, deploying the MATLAB function fmincon [59–62], to identify the optimum position of the n+1 breakpoints and the coefficients of each line segment. fmincon uses an algorithm that minimizes the Sum of Square Errors (SSE).
  $$\begin{array}{c} \Delta_{w}\left(t\right)=\{\begin{array}{cc} A_{1}t & 0<t\leq T_{1}\\ A_{2}t+B_{2} & T_{1}<t\leq T_{2}\\ A_{3}t+B_{3} & T_{2}<t\leq T_{3}\\ ...............................\\ ...............................\\ ...............................\\ ...............................\\ A_{n}t+B_{n} & T_{n-1}<t\leq T_{n} \end{array} \end{array}$$ (12)
  where *B*_2_ = *A*_1_*T*_1_, *B*_3_ = *A*_2_*T*_2_ + *B*_2_, ………, *B*_*n*_ = *A*_*n*−1_*T*_*n*−1_ + *B*_*n*−1_.
Step 5 Use the coefficients *A*_1_, *A*_2_, ….., *A*_*n*−1_, *A*_*n*_ generated using the optimization method.
Step 6 Use the coefficients in Eq 12 to reconstruct the Δ_*w*_ profile.
Step 7 Using Eq 6 reconstruct the APD profile using the Δ_*w*_ profile.
Step 8 The APD reconstruction is successful.

### Modeling dynamic properties

#### Parameterization of the Fabbri model

A similar methodology as the RM model [42] incorporating the electrophysiology of ionic current perturbation is needed to be performed. This study focuses on the simulation of the Fabbri model [6] of the human SAN cell by varying the maximum funny current block, *I*_*f*_, from 0 to 1. Eleven different APD values corresponding to equally spaced *I*_*f*_ values ranging from 0 to 1 are tabulated to investigate the relationship between the APD and the *I*_*f*_ blockade ($I_{f_{b}}$).

Table 5 defines the complexity levels of the fit-type equations. PL3, PL4 and PL5 represent the number of linear piecewise equations required for linear piecewise fitting. PL3 uses 3 linear segments for piecewise fitting. PL4 and PL5 utilize 4 and 5 linear segments for linear piecewise fitting. We use optimization method [55–58] in Fig 4 to get the location of the breakpoints for a linear piecewise polynomial. *A*_*i*_ in these equations are considered as the coefficients of the linear piecewise equations where i = 1, 2, 3,……, n as in Eq 12.

**Table 5:** General expression of various fitting functions.

Fit-type	Equation
PL3	$A_{i}\left(I_{f_{b}}\right)=\{\begin{array}{cc} p_{10}+p_{11}\cdot I_{f_{b}}, & \text{if}\;0\leq I_{f_{b}}<r_{1}\\ p_{20}+p_{21}\cdot I_{f_{b}}, & \text{if}\;r_{1}\leq I_{f_{b}}<r_{2}\\ p_{30}+p_{31}\cdot I_{f_{b}}, & \text{if}\;r_{2}\leq I_{f_{b}}\leq 1 \end{array}$
PL4	$A_{i}\left(I_{f_{b}}\right)=\{\begin{array}{cc} p_{10}+p_{11}\cdot I_{f_{b}}, & \text{if}\;0\leq I_{f_{b}}<r_{1}\\ p_{20}+p_{21}\cdot I_{f_{b}}, & \text{if}\;r_{1}\leq I_{f_{b}}<r_{2}\\ p_{30}+p_{31}\cdot I_{f_{b}}, & \text{if}\;r_{2}\leq I_{f_{b}}<r_{3}\\ p_{40}+p_{41}\cdot I_{f_{b}}, & \text{if}\;r_{3}\leq I_{f_{b}}\leq 1 \end{array}$
PL5	$A_{i}\left(I_{f_{b}}\right)=\{\begin{array}{cc} p_{10}+p_{11}\cdot I_{f_{b}}, & \text{if}\;0\leq I_{f_{b}}<r_{1}\\ p_{20}+p_{21}\cdot I_{f_{b}}, & \text{if}\;r_{1}\leq I_{f_{b}}<r_{2}\\ p_{30}+p_{31}\cdot I_{f_{b}}, & \text{if}\;r_{2}\leq I_{f_{b}}<r_{3}\\ p_{40}+p_{41}\cdot I_{f_{b}}, & \text{if}\;r_{3}\leq I_{f_{b}}<r_{4}\\ p_{50}+p_{51}\cdot I_{f_{b}}, & \text{if}\;r_{4}\leq I_{f_{b}}\leq 1 \end{array}$

#### State controller

**Fig 5:**
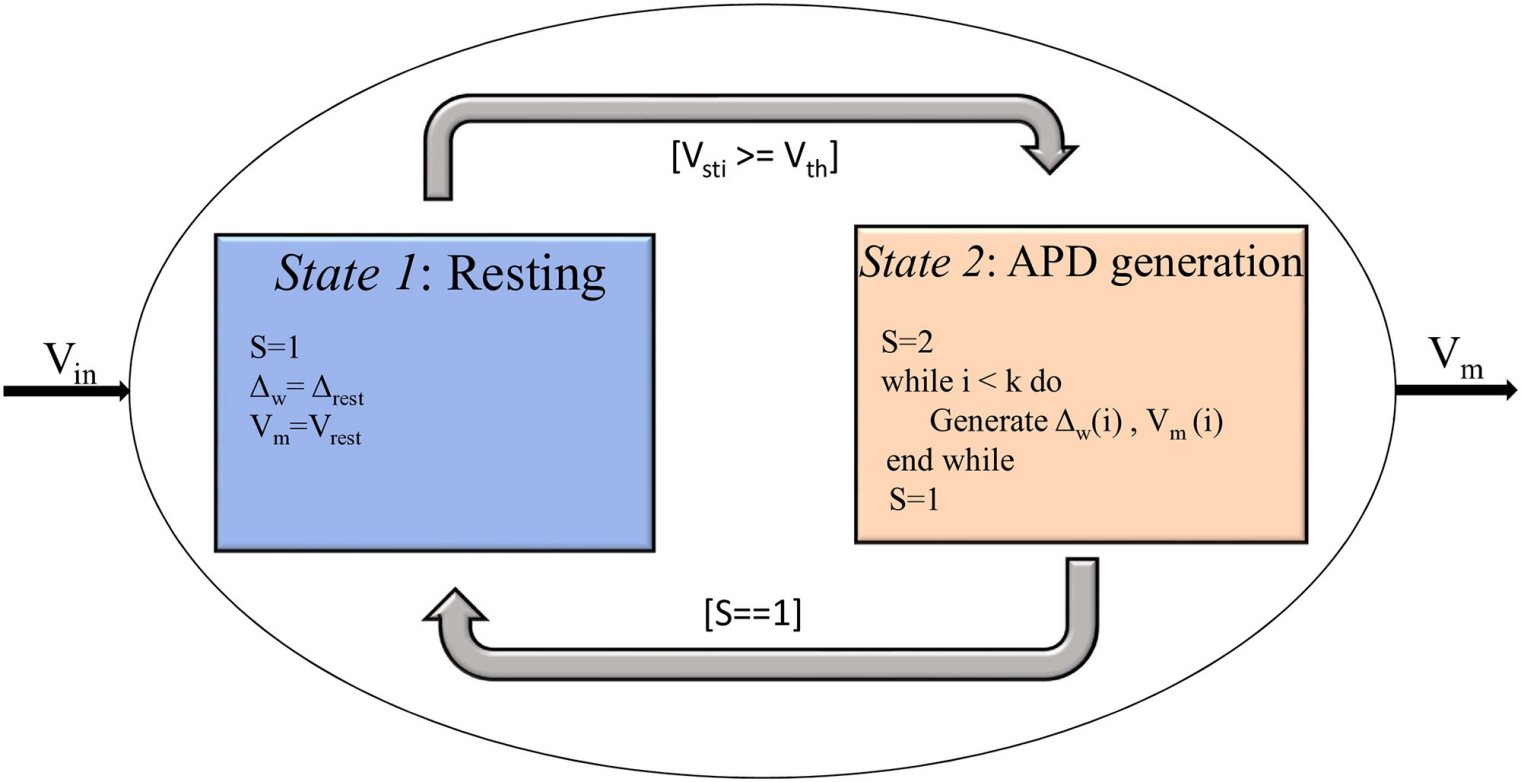
State controller of the FM model to create dynamic behavior for the FM model.

To facilitate the functioning of the 1-D and 2-D tissues using diffusion equation, we initially need to create a state controller for a single FM cell. For the current work, we designed a state controller comprising two states, i.e., State 1:resting state and State 2: APD generation state. Fig 5 shows the state controller of the FM model. Various constants and variables are required for this state controller. These include the variable, S, threshold voltage, *V*_*th*_, resting voltage, *V*_*rest*_, frequency modulating factor at rest, Δ_*rest*_, frequency modulating factor of the APD, Δ_*w*_, stimulus voltage, *V*_*sti*_ and *V*_*out*_, the voltage of the generated APD.

At State 1 (when S==1), the cell remains at rest; hence, the membrane voltage, *V*_*m*_, is at the resting voltage, *V*_*rest*_. The Δ_*w*_ is equivalent to the initial value of the Δ_*w*_ profile generated for the cell model denoted as Δ_*rest*_. The arrival of a stimulus with *V*_*sti*_ >= *V*_*th*_ causes the cell to switch to State 2, where S transitions from 1 to 2. In this state, the APD is generated for a defined number of samples, k, after which the value of S becomes 1. When S==1, the cell goes back to the resting state.

This process in turn facilitates the cell coupling in 1-D and 2-D.

#### Cell coupling using 1-D diffusion equation

The simplest computational approach to create a 1-D cell coupling is known as centered second difference [63]. The equation can be represented as

$$\begin{array}{c} \frac{d^{2}v\left(x\right)}{dx^{2}}\approx \frac{v(x+\Delta x)-2v(x)+v(x-\Delta x)}{{\left(\Delta x\right)}^{2}} \end{array}$$ (13)

This formulation in Eq 13 can be justified by combining the Taylor Series expansion for *v*(*x* + Δ*x*) and *v*(*x* − Δ*x*) (Strauss [64]). Consider the mesh size of the spacial variable as Δ*x*, then the *j*^*th*^ element of the array, v, *v*_*j*_ is of the value v for *x* = *j*Δ*x* represented by the equation below:

$$\begin{array}{c} \frac{d^{2}v}{dx^{2}}\approx \frac{\left(v_{j+1}-2v_{j}+v_{j-1}\right)}{{\left(dx\right)}^{2}} \end{array}$$ (14)

Using the expansion in Eq 14, each cell can be coupled together along with a diffusion constant D to form D(*d*^2^*v*/*dx*^2^), which can be approximated as in Eq 15.

$$\begin{array}{c} D(\frac{d^{2}v}{dx^{2}})\approx D\frac{\left(v_{j+1}-2v_{j}+v_{j-1}\right)}{{\left(dx\right)}^{2}} \end{array}$$ (15)

For simplicity of computation, Δ*x* is considered as 1. So Eq 15 can be represented in Eq 16.

$$\begin{array}{c} D(\frac{d^{2}v}{dx^{2}})\approx D\left(v_{j+1}-2v_{j}+v_{j-1}\right) \end{array}$$ (16)

**Fig 6:**
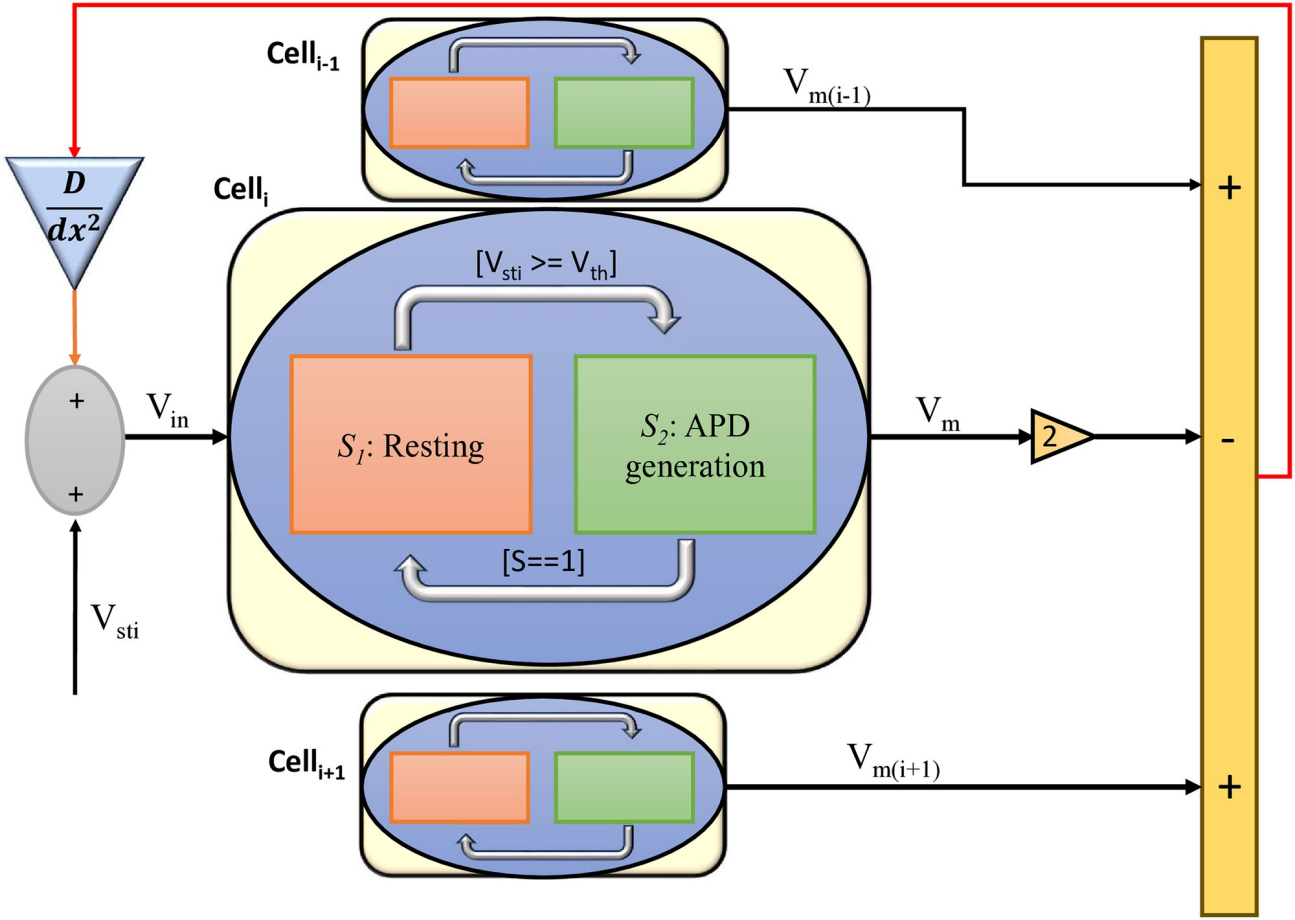
The potential design for connecting FM cells in a 1-D tissue. The detailed FM state controller is shown for the *i*^*th*^ cell. D is the diffusion coefficient. *dx* is the distance between the neighboring cells.

Fig 6 illustrates the design for connecting 1-D cells using the FM model. Each cell comprises the state controller, connected using the diffusion equations discussed in Eq 15. Similarly the state controller can be adapted to suit to the demands of the 2-D tissue in Eq 20.

#### Cell coupling using 2-D diffusion equation

The following approach can be adapted to form a 2-D cell with a mesh size of Δ*x* and Δ*y* where Δ*x* is the mesh size in the x-axis and Δ*y* is the mesh size in the y-axis. For ease of computation, both the mesh sizes are considered the same, i.e., Δ*x* = Δ*y*.

The two-dimensional diffusion equation can be represented as

$$\begin{array}{c} \frac{\partial^{2}v}{\partial x^{2}}+\frac{\partial^{2}v}{\partial y^{2}}∼\frac{v(x+\Delta x)-2v(x)+v(x-\Delta x)}{{\left(\Delta x\right)}^{2}}+\frac{v(y+\Delta y)-2v(y)+v(y-\Delta y)}{{\left(\Delta y\right)}^{2}} \end{array}$$ (17)

Consider the mesh sizes of the spacial variables to be Δ*x* and Δ*y*, then the *i*^*th*^ and *j*^*th*^ element of the array, *v*_*i*_ is of the value v for *x* = *i*Δ*x* and *v*_*j*_ is of the value v for *y* = *j*Δ*y*. Hence, we get Eq 18,

$$\begin{array}{c} \frac{\partial^{2}v}{\partial x^{2}}+\frac{\partial^{2}v}{\partial y^{2}}∼\frac{v_{i+1,j}-2v_{i,j}+v_{i-1,j}}{{\left(\Delta x\right)}^{2}}+\frac{v_{i,j+1}-2v_{i,j}+v_{i,j-1}}{{\left(\Delta y\right)}^{2}} \end{array}$$ (18)

Using Eq 18 along with the diffusion constant, D, we get Eq 19.

$$\begin{array}{c} D(\frac{\partial^{2}v}{\partial x^{2}}+\frac{\partial^{2}v}{\partial y^{2}})\approx D\frac{\left(v_{i+1,j}-2v_{i,j}+v_{i-1,j}\right)}{{\left(\Delta x\right)}^{2}}+D\frac{\left(v_{i,j+1}-2v_{i,j}+v_{i,j-1}\right)}{{\left(\Delta y\right)}^{2}} \end{array}$$ (19)

As mentioned earlier, for simplicity of calculation, Δ*x* = Δ*y* = 1. By making this adjustment, we get Eq 20,

$$\begin{array}{c} D(\frac{\partial^{2}v}{\partial x^{2}}+\frac{\partial^{2}v}{\partial y^{2}})\approx D\left(v_{i+1,j}+v_{i-1,j}-4v_{i,j}+v_{i,j+1}+v_{i,j-1}\right) \end{array}$$ (20)

#### Introduction of cellular dysfunctions to the 2-D tissues

In a 2-D tissue, cellular dysfunctions can be introduced when the input voltage, *V*_*in*_ (refer Fig 5) introduced to a cell or a set of cells is lesser than the resting voltage, *V*_*rest*_. This helps us to comprehend the alterations in the wavefront propagations which will be illustrated and tabulated in the results section.

### Overview of the methods

Overall, this section deals with how the FM model attempts to replicate the APs of the detailed model along with introducing some dynamic properties like parameterization, state controller and cell coupling. Further dynamics properties like the S1-S2 protocol, APD restitution and others will be discussed only as a future scope in this paper. The upcoming section will give a gimplse of how the AP replication will be done on an FPGA.

## Proposed FPGA friendly implementation

**Fig 7:**
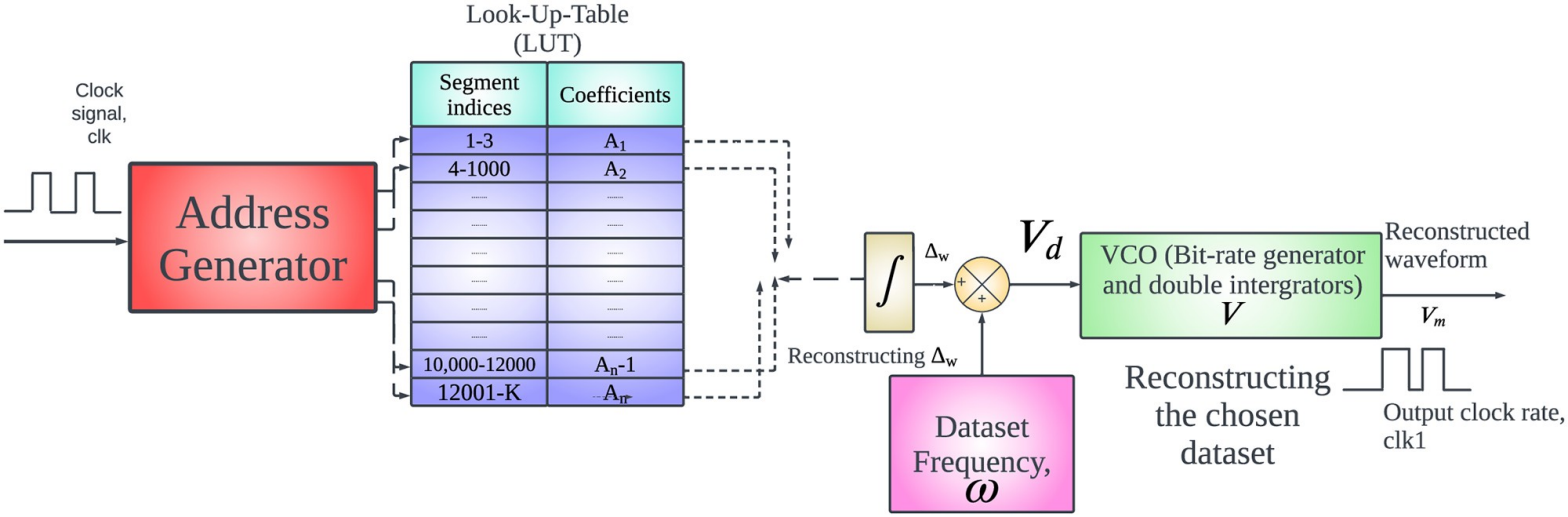
Potential FPGA implementation of the FM model.

Fig 7 shows the schematic of how the FM model can be replicated on an FPGA. It comprises of a address generator where the clock signal, clk is introduced. The address from the address generator matches with the corresponding coefficient present in the Look-Up-Table (LUT). The corresponding coefficients from each index is integrated to reconstruct the Δ_*w*_ profile. These are added with the dataset frequency, *ω*. These are introduced to a VCO which has a bit-rate generators and a double integrators to reconstruct the required waveshape, *V*_*m*_ using a varied clock rate, clk1.

## Cell models used for the novel FM model

The initial biophysical models reconstructed using the FM waveshape generation methodology are the Beeler-Reuter (BR) [5], Fenton Karma (FK) [3], FitzHugh Nagumo (FHN) [2], O’Hara [7] and Fabbri et al. [6] models. The aim is to reconstruct the APDs of these models with high precision and fidelity using the proposed FM model.

The Beeler Reuter (BR) [5] model describes the ventricular cells in generic form. This model comprises 8 non-linear ordinary differential equations (ODEs). Six equations capture the gated channel’s state. The other two equations show the intracellular *Ca*^2+^ concentration and membrane voltage (*V*_*m*_). The slow inward current (*i*_*s*_) in the Beeler Reuter (BR) model emphasizes the plateau region formed by calcium ions in non-pacemaker cells. This model includes four distinct currents within its equations: 1) the initial fast current, *I*_*Na*_; 2) a slow inward current due to calcium ions; 3) a time-activated outward current, *I*_*x*1_; and 4) a time-dependent potassium current, *I*_*k*1_.

The Fenton Karma (FK) [3] model is a simplified version of the Beeler Reuter (BR) [5] model. It comprises three non-linear ODEs. The three variables in the model are the trans-membrane potential (*u*) and two gating variables (*v* and *w*).

The Fitzhugh Nagumo (FHN) [2] model is a simplified version of the Hodgkin-Huxley (HH) model. It comprises two variables, i.e., the excitation variable, v and the recovery variable, u. Additionally, the FHN model introduces a stimulus current (I).

The Fabbri model [6] represents the human sinoatrial node (SAN) cell, commonly known as the pacemaker cell. This model comprises 33 ordinary differential equations (ODEs), with the first ODE describing the AP of the SAN cell. It visualizes and tabulates the relationship between *I*_*f*_ block and the APD variation, which the proposed FM model aims to replicate.

The O’Hara [7] model represents a human ventricular cell. This model comprises 49 ODEs. Jacob et al. [50] used this cell model to create a state controller for the RM. It was utilized to create a 1-D tissue using MATLAB. Using the FM model, 1-D and 2-D tissue models are created by reconstructing the O’Hara [7] model’s action potential duration (APD).

The APDs of these models require simultaneous ODEs. Solvers that take more computational space are required to implement these models on an FPGA. It can lead to practical limitations in generating many cells in real-time. The proposed FM model also aims to overcome these issues.

## Numerical implementations and simulations

The concept of the proposed model was simulated in SIMULINK R2022b. The test signals in the reference waveshape section were done using MATLAB R2022b. Later simulations of the reconstructions of the detailed models like Beeler-Reuter (BR), Fenton Karma (FK), FitzHugh Nagumo (FHN), Fabbri et al. [6] models. The O’Hara [7] cell models were also done on MATLAB R2022b software in the later section. The simulations were conducted on a Windows 10 PC with an Intel Core i7 3 GHz processor. It specifically focuses on determining the duration of the APDs. After this stage, the novel FM model was implemented.

## Results

In this section, we present our findings on 5 different biophysical models. We apply the FM methodology to 4 biophysical models to validate the applicability. Afterward, we show some dynamic properties of the FM cell model, which facilitates us re-emulating the tissue models in 1-D and 2-D. The tissue models capture some dynamic properties using the state controller. A more detailed analysis of other dynamic properties like restitution and refractory period must be addressed.

### Waveshape generation

#### Reconstruction of frequency modulation factor (Δ_*w*_) plots for biophysical models

**Fig 8:**
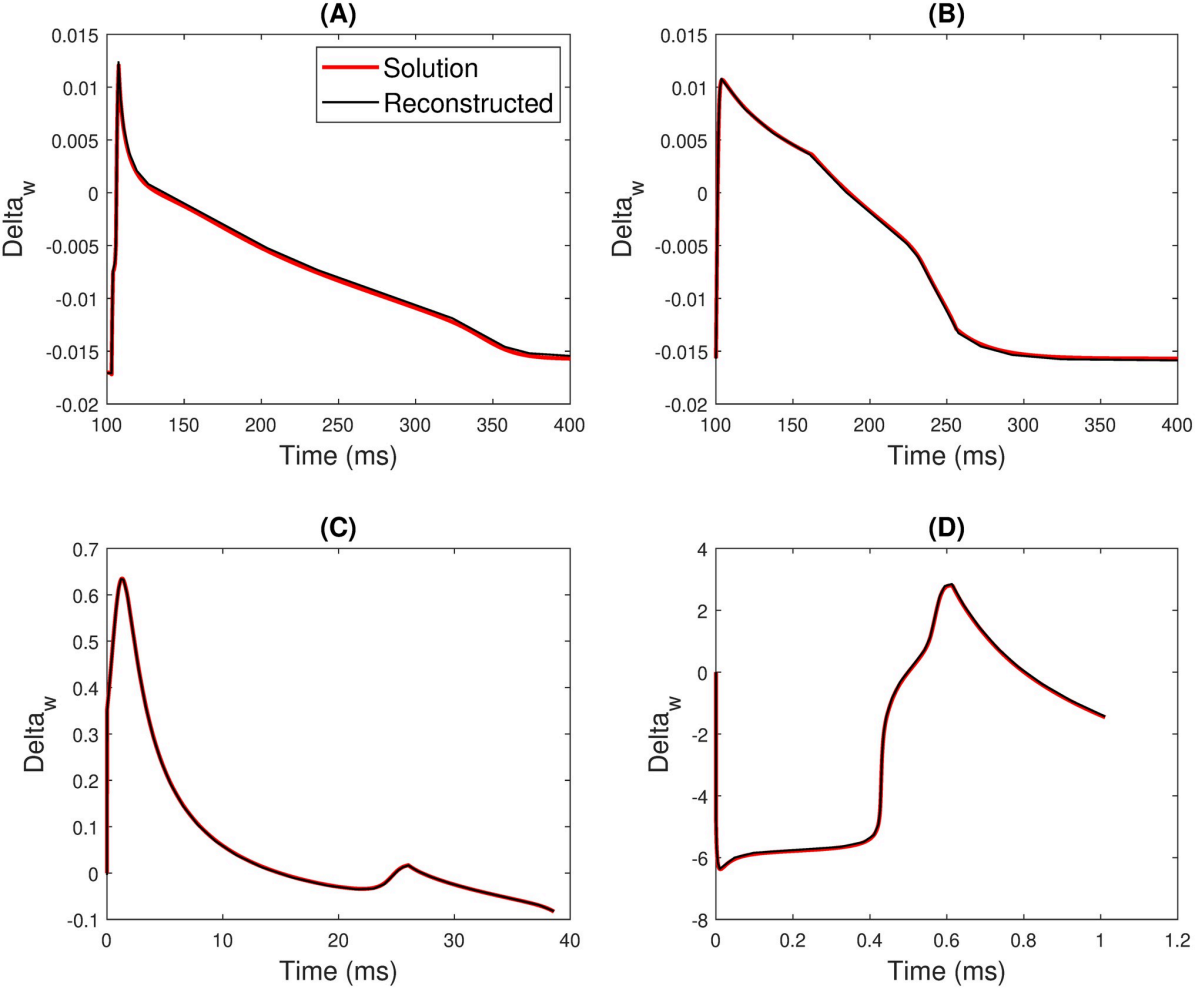
Plots of Δ_*w*_ and its corresponding reconstruction using the optimization method for (A) Beeler-Reuter(BR) [5] model (B) Fenton Karma (FK) [3] model (C) FitzHugh Nagumo (FHN) model and (D) Fabbri [6] model.

**Beeler Reuter (BR) model**. Fig 8(A) depicts the Δ_*w*_ waveshape extracted from BR model. The reconstruction of this plot utilizes 18 breakpoints for a dataset of 30000 values using linear equations. Post the optimization method, 17 linear polynomial segments were obtained (see Table 6). Fig 8(A) illustrates the Δ_*w*_ waveshape and its reconstruction for the BR model.

**Table 6:** RMSE and *R*^2^ values of the Δ_*w*_ and APD reconstructions using the optimization method for the cell models.

Models	No of segments	No of datapoints	RMSE of Δ_*w*_	*R*^2^ of Δ_*w*_	RMSE of APD	*R*^2^ of APD
BR model	17	30000	0.0002	0.9983	2.6470	0.9952
FK model	15	30000	0.0001	0.9998	0.0058	0.9998
FHN model	31	3861	0.0007	1.0000	0.0172	0.9998
Fabbri model	29	10400	0.0338	0.9999	0.5033	0.9995

**Fenton Karma (FK) model**. Fig 8(B) shows the Δ_*w*_ waveshape and its corresponding reconstructions. The reconstruction involves 16 breakpoints for a dataset of 30000 values, resulting in 15 linear piecewise equations after optimization (refer to Table 6).

**FitzHugh Nagumo (FHN) model**. The FHN model shows the APD of a neuronal cell. Hence, the Δ_*w*_ wave shape for the FHN model (Fig 8(C)) differs in a big way when compared to BR and FK models. Thrity-two breakpoints were assumed for a dataset of 3861 values. Post-optimization, 31 linear segments were obtained (Table 6). Fig 8(C) also includes the reconstruction of the Δ_*w*_ plot incorporating the mentioned parameters.

**Fabbri model**. The Fabbri [6] model emulates a pacemaker called the SAN cell. Hence, this model’s Δ_*w*_ waveform varies from the other three cell models. For data of 10400 values, 29 segments were required to attain an optimized reconstruction of the Δ_*w*_ waveshape (Table 6). Fig 8(D) portrays the reconstruction utilizing the optimized linear equations.

Table 6 tabulates the Root Mean Square Error (RMSE) and *R*^2^ error values. The *R*^2^ values are consistently above 0.99 across all the models. However, the BR model quantifies a relatively higher RMSE than other reconstructions. For the FK and FHN models, the RMSE falls below 0.0003.

#### Reconstruction of the Action Potential Duration (APD) using frequency modulation factor (Δ_*w*_) plots

Upon successfully reconstructing the Δ_*w*_ plots for the mentioned biophysical models, assessing their ability to accurately reconstruct these cell models’ Action Potential Duration (APD) becomes crucial. It is pivotal to observe that the APDs and Δ_*w*_ waveform contain an equal number of data points, as Δ_*w*_ is derived from the APDs of the cell models. Theoretical Background explains Eq 6, which incorporates the frequency modulating factor, Δ_*w*_, with the Action Potential Duration’s (APD’s) frequency, *ω*.

**Fig 9:**
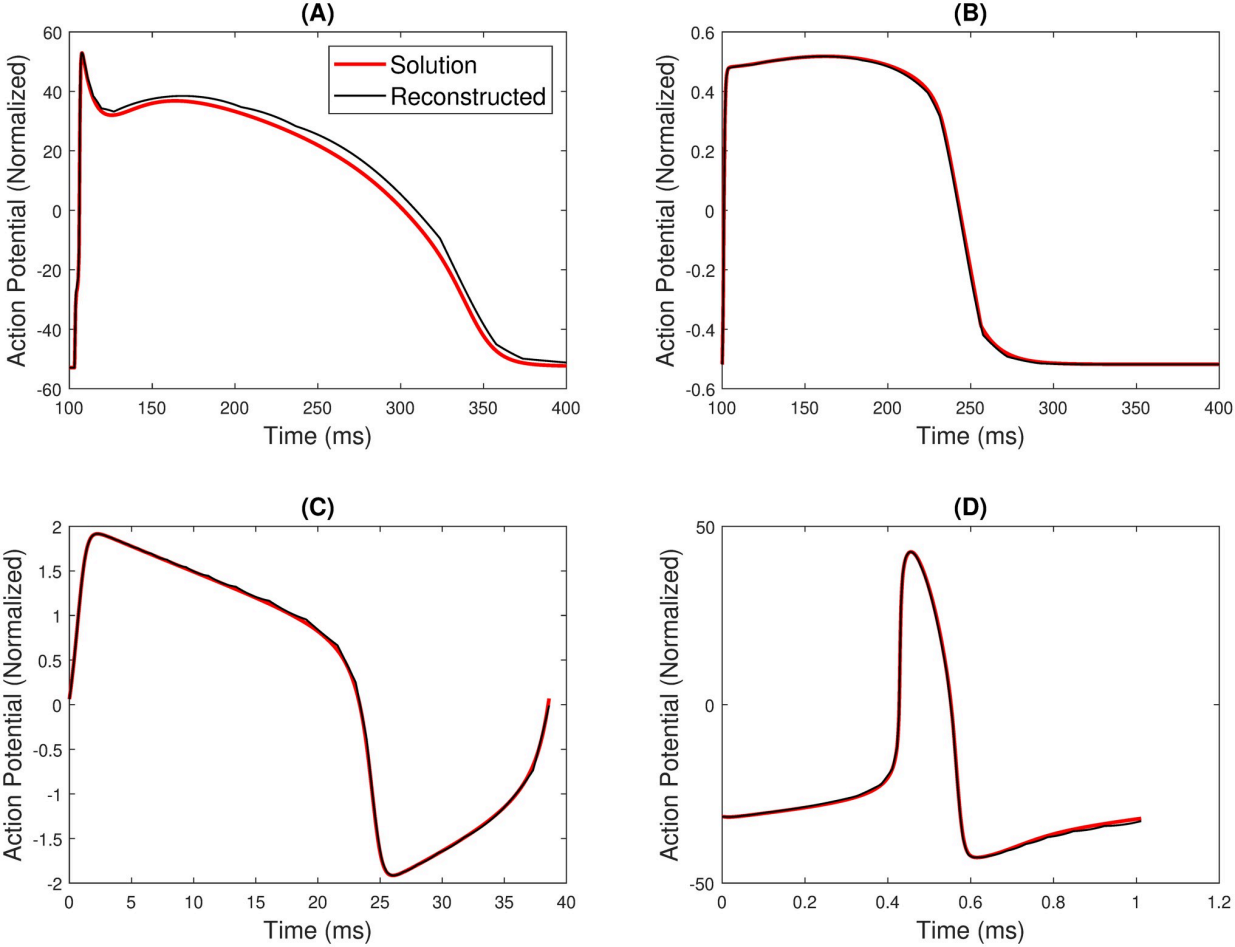
APD waveshape and its corresponding reconstruction for (A) Beeler-Reuter(BR) model (B) Fenton Karma (FK) model (C) FitzHugh Nagumo (FHN) model and (D) Fabbri [6] model.

*Beeler Reuter (BR) model*. Fig 9(A) presents the reconstructed APD of the BR model from the corresponding Δ_*w*_ reconstruction. Despite the slight quantitative offset observed after reaching the peak value, the APD closely resembles the solution.

*Fenton Karma (FK) model*. Fig 9(B) showcases the reconstruction of the APD from its Δ_*w*_ plot illustrated in Fig 8(B). A remarkable similarity is observed when the reconstructed APD is compared to the APD of the detailed model. Notably, the number of data points for BR and FK models is 30000.

*FitzHugh Nagumo (FHN) model*. The reconstructed APD of the FHN model is shown in Fig 9(C) which is generated using a 31-segment linear polynomial. The *R*^2^ is found to be more than 0.999, and the RMSE is found to be approximately 0.0172.

*Fabbri model*. The reconstructed APD of the Fabbri [6] model, representing a SAN or the pacemaker cell, is shown in Fig 9(D). It has *R*^2^ of more than 0.999 and portrays a very low RMSE of approximately 0.5.

Interestingly, despite having similar data points, the BR model replication exhibits higher RMSE than the FK model. The FK model portrays the lowest RMSE (see Table 6) among all the APD reconstructions done. All the cell model APD profiles exhibit a *R*^2^ of above 0.99.

This investigation sheds light on how bankable the FM model is for reconstructing APDs of various cell models. Moreover, the tabulations and illustrations emphasize how effectively the FM model captures the essential characteristics of the original APD profiles.

### Modeling dynamic properties

#### Parameterization of the novel FM model

**Fig 10:**
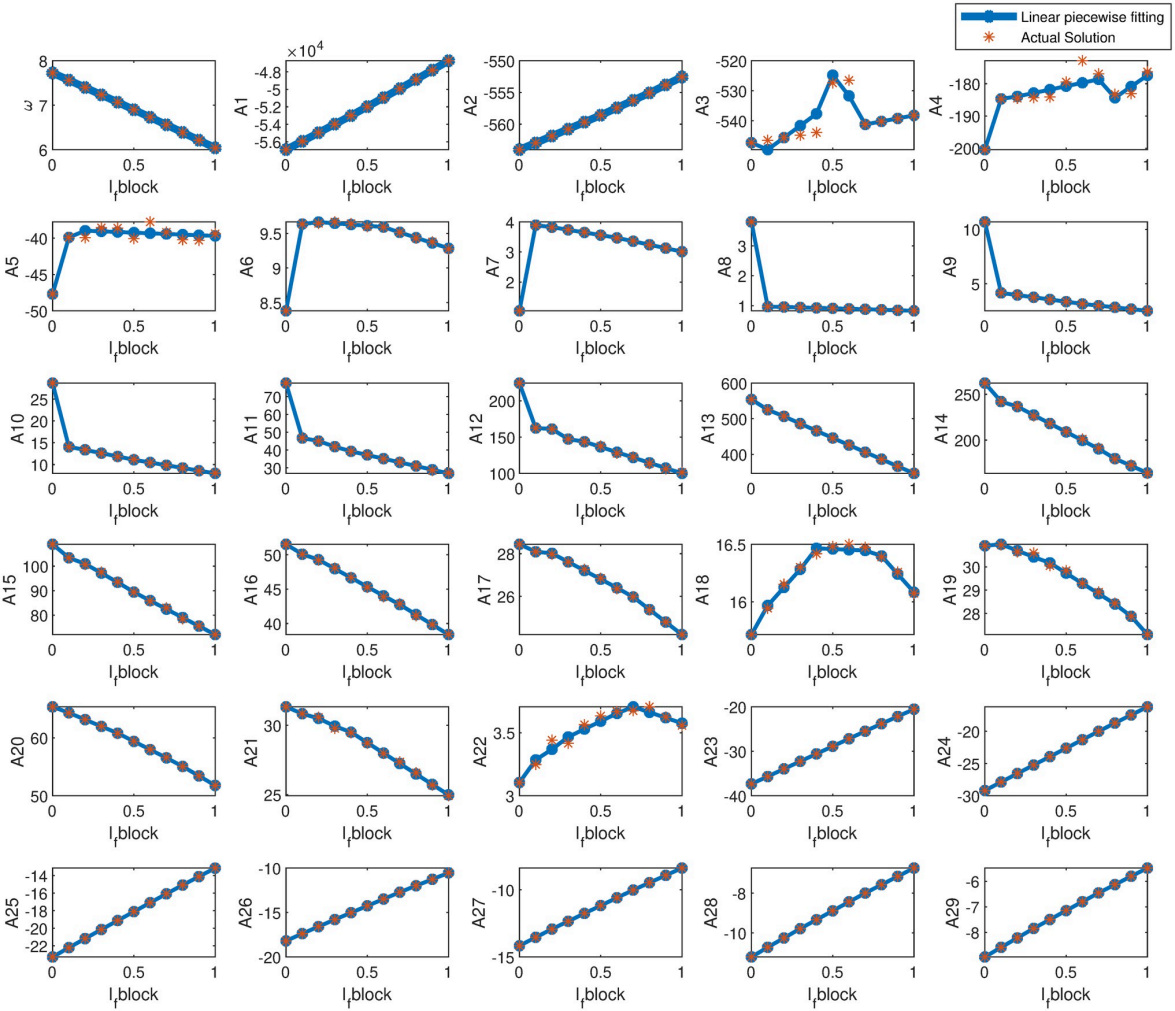
Minimum piecewise linear fits for the polynomial values of Δ_*w*_ of the sinoatrial node cell at different levels of *I*_*f*_ blockade. *ω* is the frequency of the APDs replicated. Δ_*w*_ is divided into 29 different coefficients comprising A1, A2,…., A29.

For recreating the Δ_*w*_ waveshapes of the Fabbri [6] model using this novel methodology, we take 30 breakpoints, which allow us to formulate 29 linear segments with coefficients A1, A2,…., A29. Fig 10 illustrates the profiles of each polynomial value when subjected to varying $I_{f_{b}}$ in increments of 0.1. Astonishingly, the trajectories of these polynomial values are linear. Hence, an attempt is made to derive closed-form linear equations that describe the alterations of the polynomial values and the frequency of the dataset, *ω*, for varying $I_{f_{b}}$. These equations enable the reconstruction of the APDs for an arbitrary $I_{f_{b}}$.

Table 7 tabulates RMSE and *R*^2^ of the reconstructed APDs utilizing the Δ_*w*_ waveshape generated from the fit-type equations to the original APD solution. The RMSE values for the reconstructed Δ_*w*_ vary from approximately 0.016 to 0.05. The highest *R*^2^ for the reconstructed Δ_*w*_ is found for $I_{f_{b}}=0.6$ where *R*^2^ = 1. The RMSE at $I_{f_{b}}=0.6$ is also the lowest. RMSE of reconstructed APDs vary approximately 0.2 to 0.7. The highest *R*^2^ for the reconstructed APD is found for $I_{f_{b}}=0.2$ and $I_{f_{b}}=0.6$ where *R*^2^ = 0.9999. However, the RMSE for $I_{f_{b}}=0.2$ is relatively higher than that of $I_{f_{b}}=0.6$. The RMSE is highest, and the *R*^2^ is the lowest for $I_{f_{b}}=0.9$ where RMSE = 0.7258 and *R*^2^ = 0.9989. The *R*^2^ for the reconstructed APD varies between 0.9989 to 0.9999. This tabulation showcases that these fit-type equations can effectively replicate the Δ_*w*_ and the AP characteristics when the $I_{f_{b}}$ is altered in increments of 0.1 from 0 to 1.

**Table 7:** RMSE and *R*^2^ values of the Δ_*w*_ and APD reconstructions using the fit-type equations for different values of *I*_*f*_ blockade.

$I_{f_{b}}$	RMSE of Δ_*w*_	*R*^2^ of Δ_*w*_	RMSE of APDs	*R*^2^ of APDs
0	0.0480	0.9998	0.5507	0.9995
0.1	0.0244	1.0000	0.2905	0.9998
0.2	0.0290	0.9999	0.2678	0.9999
0.3	0.0380	0.9999	0.4593	0.9996
0.4	0.0515	0.9998	0.7129	0.9990
0.5	0.0298	0.9999	0.3103	0.9998
0.6	0.0159	1.0000	0.1978	0.9999
0.7	0.0276	0.9999	0.3095	0.9998
0.8	0.0429	0.9998	0.6578	0.9991
0.9	0.0476	0.9998	0.7258	0.9989
1.0	0.0302	0.9999	0.4060	0.9996
0.001	0.0380	0.9999	0.4298	0.9997
0.175	0.0642	0.9997	0.8980	0.9985
0.250	0.0258	1.0000	0.2042	0.9999
0.38	0.0515	0.9998	0.6803	0.9991
0.45	0.0247	0.9998	0.0516	0.9992
0.567	0.0389	0.9996	0.9380	0.9982
0.657	0.0386	0.9999	0.6141	0.9992
0.750	0.0358	0.9982	0.5891	0.9993
0.875	0.0346	0.9999	0.5360	0.9994
0.950	0.0445	0.9998	0.6404	0.9991
0.998	0.0123	1.0000	0.1687	0.9999

For evaluating how consistent reconstructed Δ_*w*_ and APD plots are when assigning random values to the *I*_*f*_ blockade ($I_{f_{b}}$), a similar assessment was tabulated to Table 7. It also demonstrates the performance of linear fit-type equations when random values of $I_{f_{b}}$ from 0 to 1 are applied. The various values of $I_{f_{b}}$ are 0.001, 0.175, 0.25, 0.38 and others. The *R*^2^ reveal that the linear fit-type equations consistently reconstruct the Δ_*w*_ waveshape for all the attempted random values of $I_{f_{b}}$, as all of them are above 0.998 (refer Table 7). RMSE of the Δ_*w*_ replication ranges from 0.0123 to 0.0642. Notably, a surge in *R*^2^ corresponds to a decrease in RMSE, reinforcing the effectiveness of the linear fit-type equations in capturing the Δ_*w*_ characteristics for various *I*_*f*_ blockade, $I_{f_{b}}$. Table 7 also quantitatively analyzes how the random $I_{f_{b}}$ values from 0 to 1 can reconstruct the APD waveshapes. Despite a higher RMSE for the APD waveshape replication, the *R*^2^ is consistently above 0.998. The highest RMSE is at $I_{f_{b}}=0.567$, corresponding to the lowest the lowest *R*^2^,i.e., 0.9982. The RMSE for the APD reconstruction is also the lowest at $I_{f_{b}}=0.998$, corresponding to the highest *R*^2^, i.e., 0.9999. To sum up, the linear fit-type equations successfully replicate the Δ_*w*_ and AP characteristics of the SAN call not only for $I_{f_{b}}$ values in increments of 0.1 but also for arbitrary $I_{f_{b}}$ values between 0 to 1.

**Fig 11:**
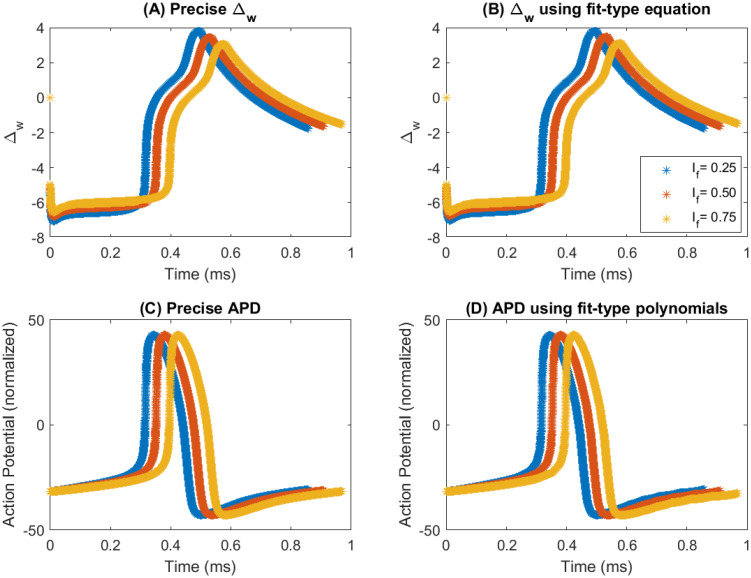
Variation of Δ_*w*_ waveshape and APD when $I_{f_{b}}$ = 0.25, 0.50 and 0.75 (A) Precise Δ_*w*_ (B) Δ_*w*_ using fit-type equations (C) Precise APD (D) APD using fit-type equations.

Fig 11(A) and 11(B) visually illustrate the alterations in the Δ_*w*_ waveshape for $I_{f_{b}}=0.25,0.5$ and 0.75. In addition to this, Fig 11(C) and 11(D) depict how the AP characteristics vary when the *I*_*f*_ blockade, $I_{f_{b}}$ varied at 0.25, 0.5, and 0.75. These figures portray how the reconstructed waveshapes closely resemble the waveshapes of the detailed model when subjected to a particular *I*_*f*_ blockade, $I_{f_{b}}$. Hence, we have conclusive evidence that the FM model can alter the AP characteristics of a SAN cell when there is an alteration in the values of *I*_*f*_ blockade, $I_{f_{b}}$.

The investigation in this subsection affirms that the FM model can accurately reconstruct the APD of the human SAN cell for any $I_{f_{b}}$ ranging from 0 to 1.

#### State controller

**Fig 12:**
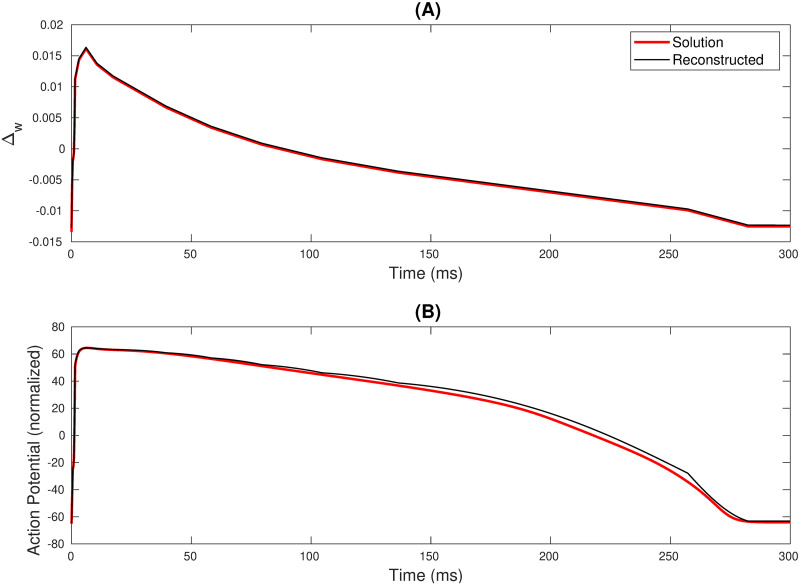
Reconstruction of the (A) the Δ_*w*_ profile and (B) the APD replication of the O’Hara [7] cell model.

This part of the work discusses creating more complexity and capability for the reconstructed AP characteristics using the FM methodology. Sehgal et al. [41] discuss the state controller for reconstructing the O’Hara [7] model. Jacob et al. [47] utilized it to create a 1-D tissue. Hence, we need to reconstruct the APD of the O’Hara [7] cell model using the FM model. Fig 12(A) and 12(B) showcase the replication of the O’Hara [7] cell model. To reconstruct this model’s Δ_*w*_ profile, we used 17 segments, i.e., 18 breakpoints for 30001 data points. The replication of the Δ_*w*_ profile is illustrated in Fig 12(A). Like any other cell model’s reconstruction, we calculated the RMSE and *R*^2^ of the reconstructed Δ_*w*_ waveshape. The RMSE of the reconstructed Δ_*w*_ profile was as low as 0.0002, and the *R*^2^ was approximately 0.9993. The APD replication is found to have low RMSE, corresponding to the high *R*^2^ value.

**Fig 13:**
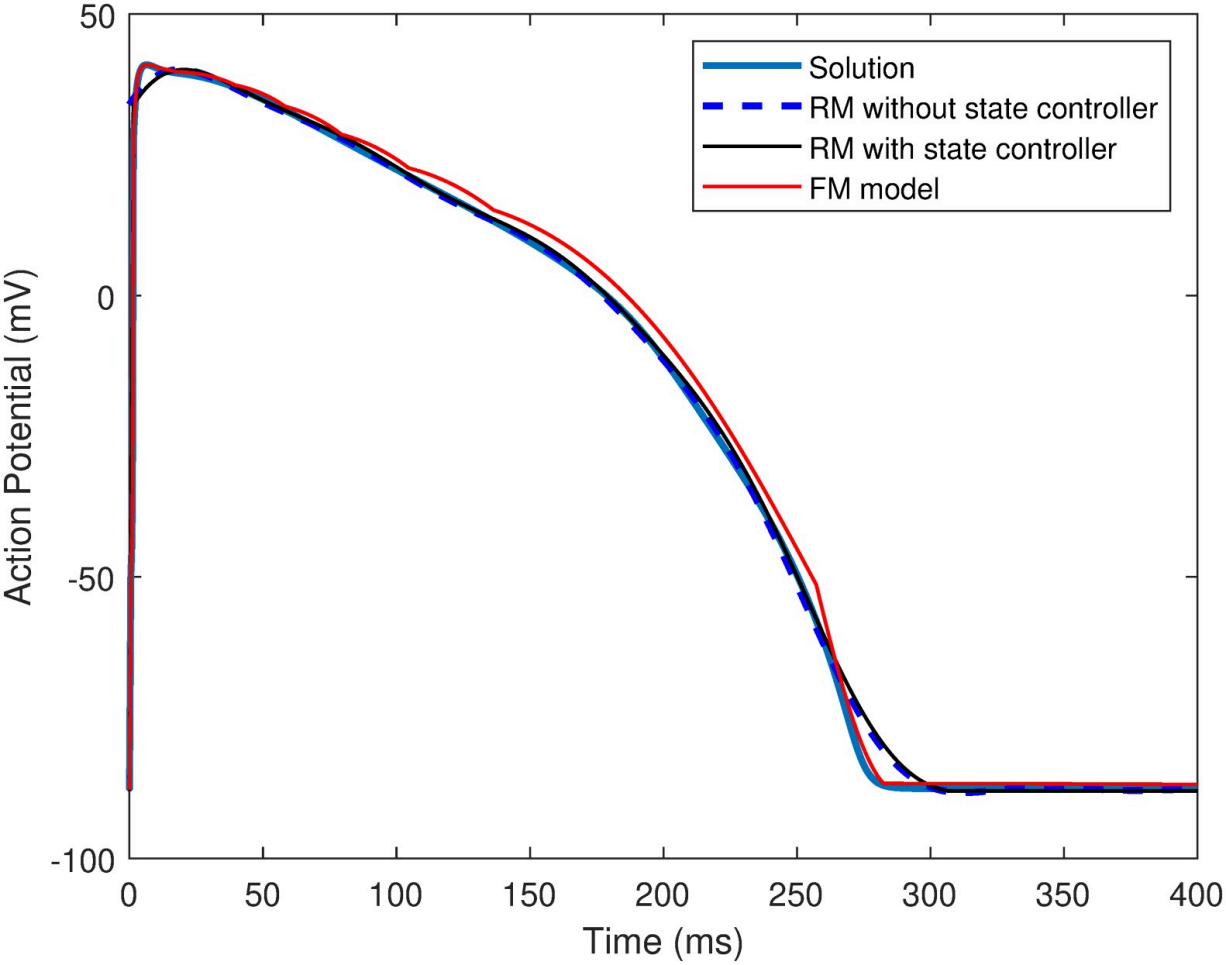
The point-to-point difference in APD reconstruction w.r.t solution for RM without state controller, RM with state controller and FM model.

Sehgal et al. [41] had three states in their state controller comprising *S*_1_: resting state, *S*_2_: Upstroke, and *S*_3_: Repolarization states. These states present in the state controller of the RM are bounded by a certain set of conditions. Fig 13 shows the point-to-point variations of the APD reconstruction of RM with and without state controller along with the replication using the FM model. Table 8 depicts the RMSE and *R*^2^ of the O’Hara [7] APD replication using the three techniques. It can be observed that the RMSE of the RM cell without a state controller is significantly high, i.e., 5.3603. This metric affects the *R*^2^ and is the lowest among the three. On utilizing RM with state controller, the RMSE dwindles significantly from 5.3603 to 2.1849, which also shoots up the *R*^2^. The FM model replication’s RMSE and *R*^2^ values are almost similar to that of the RM with the state controller.

**Table 8:** The RMSE and *R*^2^ values of O’Hara [7] model reconstructions using RM with/without the state controller and the FM model.

Method	RMSE	*R* ^2^
RM without state controller	5.3603	0.9885
RM with state controller	2.1849	0.9985
FM model	2.4601	0.9976

**Fig 14:**
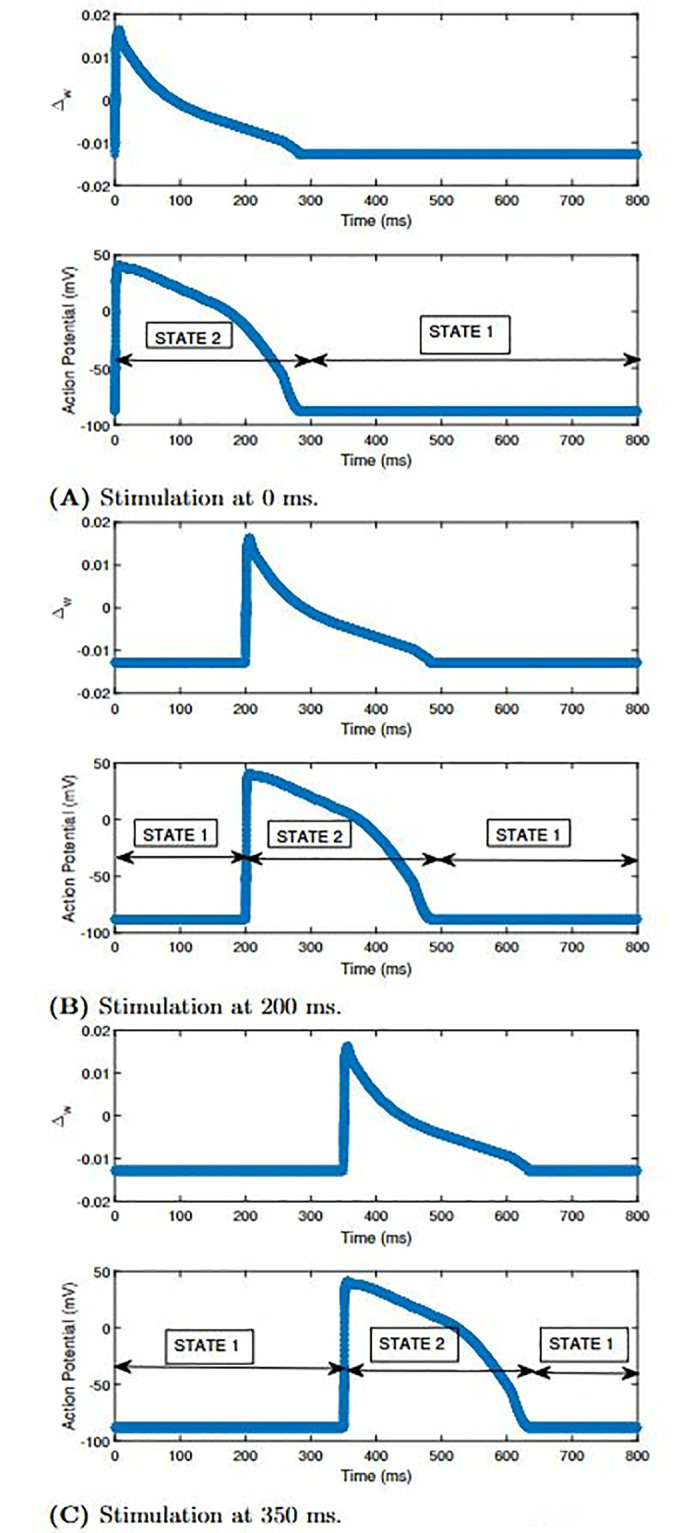
The Δ_*w*_ and APD reconstructions of the O’Hara [7] model along with the alterations in the state value in the FM state controller when the Stimulation is at (A) 0 ms (B) 200 ms and (C) 350 ms. (**A**) Stimulation at 0 ms. (**B**) Stimulation at 200 ms. (**C**) Stimulation at 350 ms.

Hence, it is imperative to know whether the state controller allows the cell stimulation at 0 ms. When the stimulus was applied at 0 ms, as depicted in Fig 14(A), S begins at 2 rather than 1. Hence, we can infer that the cell reacts to the stimulus at 0 ms. We can observe that S remains at 2 till 300 ms. After 300 ms, no stimulus is applied and cell remains at resting state. In Fig 14(B), the stimulus is applied at 200 ms for the cell. From 0 to 200 ms, S was at 1,i.e., the cell was at rest during this period. The state shifts from 1 to 2 after 200 ms, i.e., the cell has entered the APD state. The APD state is found to be ending at 500 ms when the state, S, is altered from 2 to 1. Fig 14(C) illustrates a similar waveform and state evolution as that of Fig 14(A) and 14(B) when the stimulus is applied at 350 ms. Similar observations and analysis should be done for the APD reconstructions of the FHN model.

**Fig 15:**
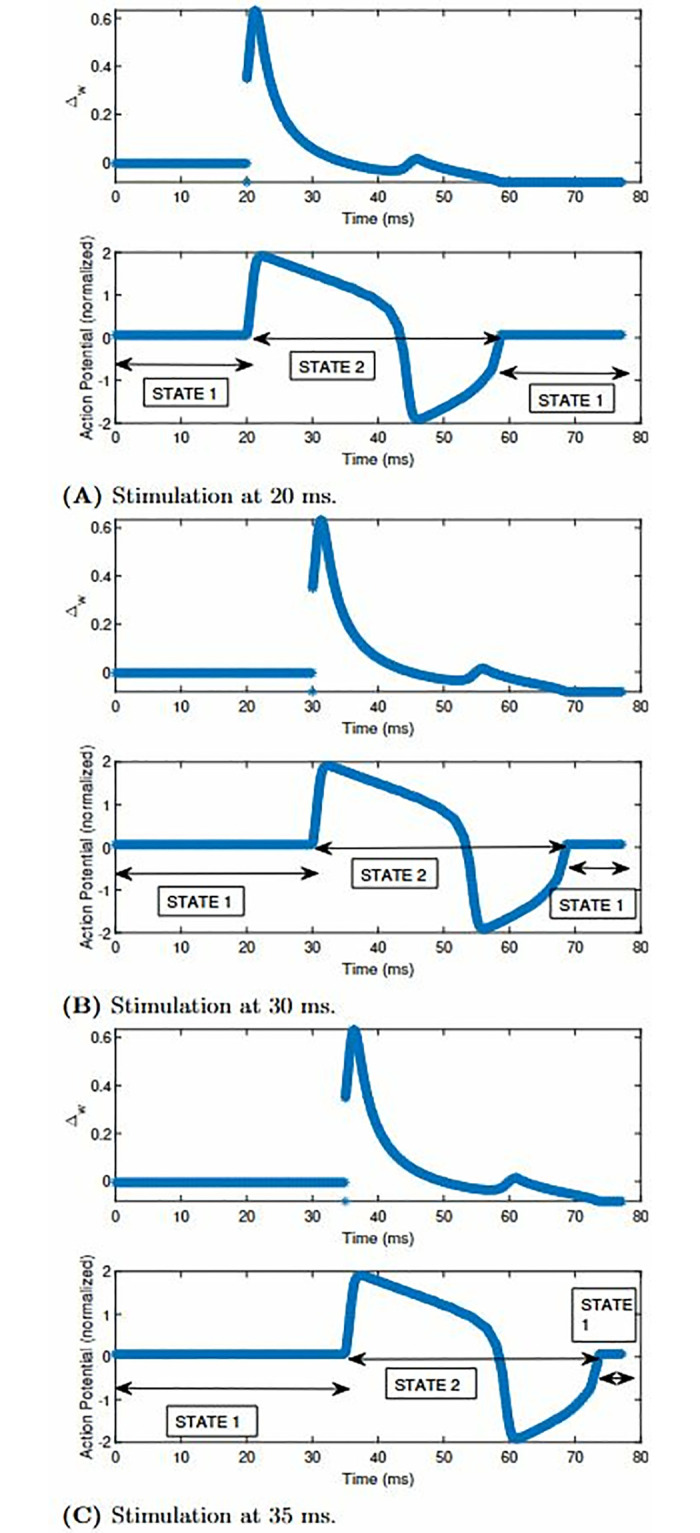
The Δ_*w*_ and APD reconstructions of the FHN model along with the alterations in the state value in the FM state controller when the stimulation is at (A) 20 ms, (B) 30 ms and (C) 35 ms. (**A**) Stimulation at 20 ms. (**B**) Stimulation at 30 ms. (**C**) Stimulation at 35 ms.

Since the reconstruction of the FHN model using the FM model began at 0 ms, we do not need to test whether the FM state controller stimulates the AP characteristics at that instant. Hence, we look to execute the state controller for the replicated APD of the FHN model at other time instants like 20 ms, 30 ms and 35 ms. Fig 15(A) showcases how the AP characteristics of the reconstructed FHN model propagates at 20 ms. From the time instants between 0 ms to 20 ms, the state value of the state controller remains at 1. After 20 ms, we can observe that S alters from 1 to 2. When the S is at 2, the APD of the FHN model is generated exactly as the reconstructed FHN model using the FM model. When the S is 2, the APD of the FHN model is generated exactly as the reconstructed FHN model as per the FM methodology. The APD propagation ends at 60 ms. Afterward, S alters itself from 2 to 1. On altering the time instant of the stimulus from 20 ms to 30 ms, we get Fig 15(B). In this case, S is 1 from 0 ms to 30 ms. After 30 ms, the APD propagates with the reconstructed characteristics of the FHN model. The propagation starts from 30 ms and ends at approximately 70 ms. The S value of the state controller alters from state 1 to 2 in this period. When the time instant is after 70 ms, S alters from 2 to 1, showing that the FM cell is at rest. To further test the state controller with this cell model, we altered the time instant of stimulation from 30 ms to 35 ms (refer Fig 15(C)). The FM cell remains at the APD state from 35 ms to approximately 75 ms. Afterward, S alters from 2 to 1, showing that after 75 ms, the FM cell remains resting.

#### Cell coupling using 1-D diffusion equation

Previously, we have implemented a state controller of the FM cell model, which creates complexity and capability for the reconstructed APDs of the cell models such as O’Hara [7] and FHN models. Fig 6 illustrates the design for connecting 1-D cells using the FM model. Each cell comprises the state controller, connected using the diffusion equations discussed in Eq 15.

**Fig 16:**
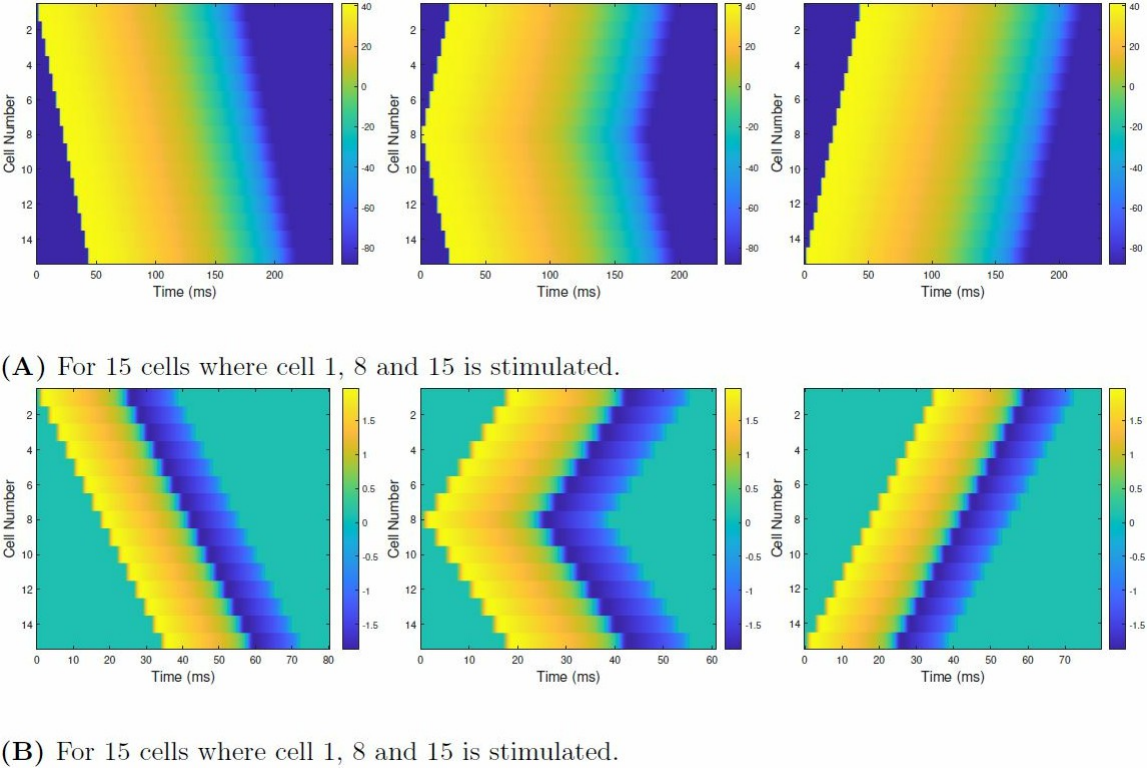
The propagation wavefront of 1-D FM model tissue comprising 15 cells for three different junctures of stimulation for (A) ventricular cell where D = 0.0625 for step time of 0.4 ms (B) a nerve cell where D = 0.00375 for step time of 0.1 ms. (**A**) For 15 cells where cell 1, 8 and 15 is stimulated. (**B**) For 15 cells where cell 1, 8 and 15 is stimulated.

Fig 16(A) and 16(B) illustrate the 1-D propagations wavefront for O’Hara [7] and FHN model where the stimulus is applied at cell 1, cell 8, and cell 15. The diffusion constant, D and step time for Fig 16(A) is 0.0625 and 0.4 ms. While for Fig 16(B), these are 0.00375 and 0.1 ms. In Fig 16(A) and 16(B), when the stimulus is applied at cell 1, the wavefront propagates from cell 1 to cell 15. When the stimulus is altered from cell 1 to 8, the wavefront propagates outwardly, i.e., from cell 8 to cell 1 and cell 8 to cell 15. When cell 15 is stimulated, the propagation wavefront evolves from cell 15 to cell 1.

#### Cell coupling using 2-D diffusion equation

Earlier, we showed the wavefront propagation of the 1-D tissue using the FM model. We will observe how a 2-D FM tissue is governed by the diffusion equation in Eq 20 for O’Hara [7] and FHN models. The cell assignment in a 2-D tissue is done in a systematic pattern. In a 15x15 2-D tissue, the cell at the top-left corner is cell 1, while the cell at the bottom-right corner is cell *N*^2^, i.e., 225, and the middle cell can be found using (*N*^2^ + 1)/2, i.e., 113. Initially, we attempted to find the propagation wavefront of a 2-D FM tissue where the stimulus is applied at 4 different junctures at (1) cell 1, (2) cell (*N*^2^+ 1)/2, (3) cell *N*^2^ and (4) cell 1 to N in the vertical direction for a 7x7,15x15 and 31x31 2-D FM tissue.

**Fig 17:**
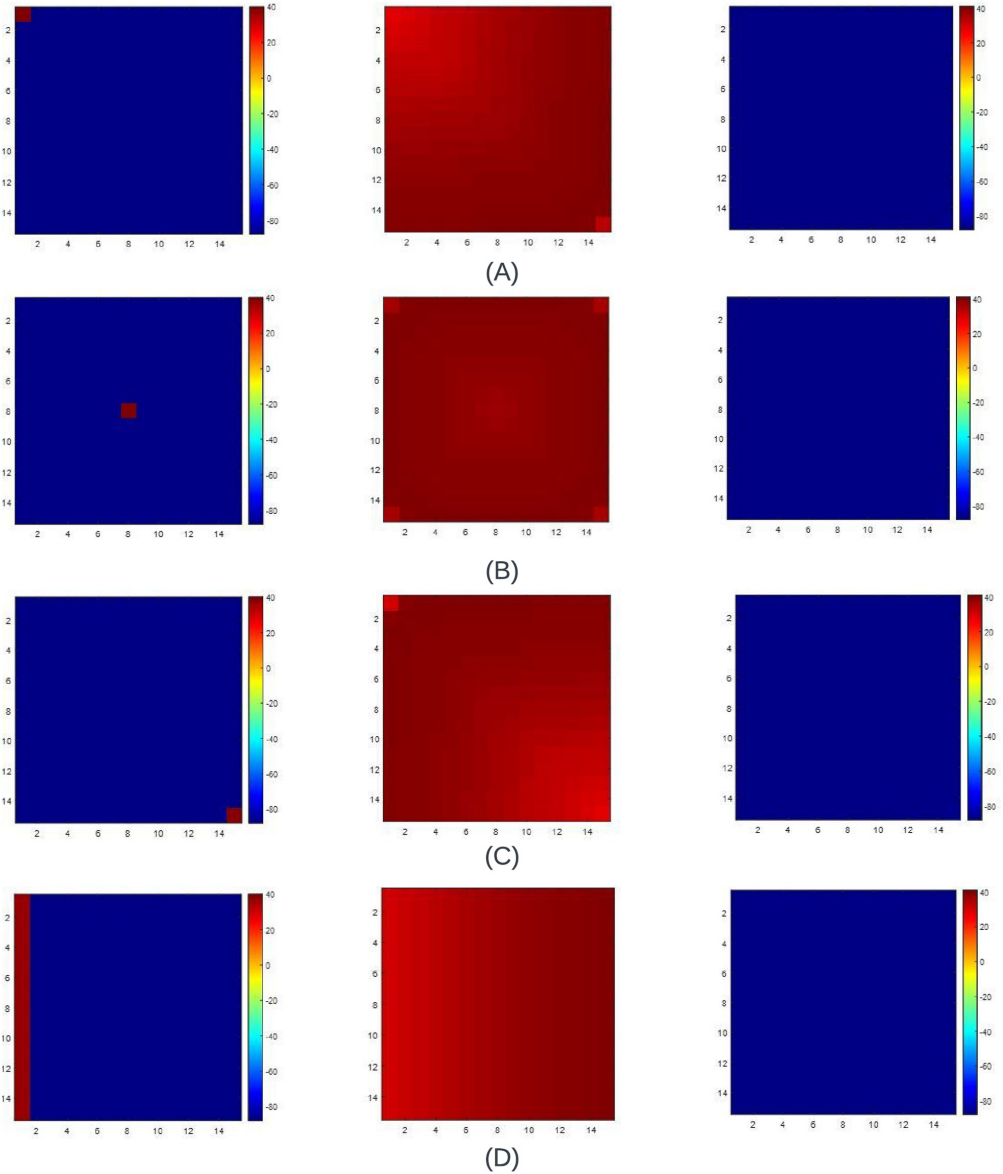
The propagation wavefront of a 15x15 2-D tissue of the O’Hara [7] model when the (A) Cell 1 is stimulated (B) Cell (*N*^2^ + 1)/2 is stimulated, i.e., Cell 113 for a 15x15 cell matrix is stimulated (C) Cell NxN, i.e., 225 is stimulated and (D) Multiple cell from Cell 1 to N is stimulated where N = 15 is stimulated. The propagation wavefront starts differently for each stimulation, but in the end, all the cells in the 2-D tissue rest when the cell or cells are stimulated to return to rest. The diffusion constant, D, used in all cases is 0.0625, and the step time is 0.4 ms.

Fig 17(A)–17(D) showcase the propagation wavefront when the stimulus is exerted on a 2-D tissue comprising 15x15 cells. The diffusion constant and step time are 0.0625 and 0.4 ms. When cell 1 is stimulated, the propagation wavefront evolves, as shown in Fig 17(A). The wavefront propagates outwardly, affecting all the adjacent cells from cell 1 to cell *N*^2^ = 225. When cell 1 is found to go to the resting state, we can observe that all the cells within the 2-D tissue gradually go to the resting state. When the stimulus exerted is shifted from cell 1 to cell (*N*^2^ + 1)/2, i.e., 113, we get the wavefront to propagate as in Fig 17(B). The wavefront propagates outwardly in all directions, affecting the surrounding cells within the 2-D tissue. As cell 113 is found to be at resting condition, all the cells are within the 2-D tissue. Later, we look to shift the stimulus from cell (*N*^2^ + 1)/2 to cell *N*^2^, i.e., 225 in this case. The illustrations are displayed in Fig 17(C). The wavefront propagates opposite to the illustration showcased in Fig 17(A). The propagation begins from cell 225 to cell 1, diagonally affecting the adjacent cells. As cell 225 goes to rest, all the cells within the 2-D FM tissue gradually do so. Afterward, as shown in Fig 17(D), we stimulated cells 1 to 15 together in the vertical direction. In this case, the wavefront propagates linearly in the vertical direction. When cells 1 to 15 go to the resting state, we can observe that all the adjacent cells tend to go to the resting state. Similar observations were found for the 7x7 and 31x31 2-D FM tissues.

Various parameters are required to justify working a 7x7, 15x15 and 31x31 2-D FM tissue. These are

1. Peak time at cell 1, $T_{p_{1}}$: The time at which cell 1 reaches the value of the maximum membrane potential. It is measured in milliseconds (ms).
2. Mid-cell peak time, $T_{p_{mid}}$ (ms): The time at which the cell (*N*^2^+1)/2 reaches the maximum membrane potential.
3. Peak time at cell *N*^2^, $T_{p_{NN}}$ (ms): The time at which the cell *N*^2^ reaches the maximum membrane potential.
4. Mid to cell 1 peak time, *T*_*mc*1_ (ms): The absolute difference in peak time at cell 1 to the mid-cell peak time. The equation is depicted in Eq 21.
  $$\begin{array}{c} T_{mc1}=\left|T_{p_{1}}-T_{p_{mid}}\right| \end{array}$$ (21)
5. Mid to cell NxN peak time, *T*_*mNN*_ (ms): The absolute difference in peak time at cell *N*^2^ to the mid cell’s peak time. The formulation of this parameter is shown in Eq 22.
  $$\begin{array}{c} T_{mNN}=\left|T_{p_{NN}}-T_{p_{mid}}\right| \end{array}$$ (22)
6. Relative adjacent peak time, *T*_*adj*_ (ms): The absolute difference between the peak time of the first stimulated cell and the peak time of the adjacent cells.
  $$\begin{array}{c} T_{adj}=\left|T_{p_{N}}-T_{p_{N_{near}}}\right| \end{array}$$ (23)
  where $T_{p_{N}}$ is the peak time of the initially stimulated cell and $T_{p_{N_{near}}}$ is the peak time of the nearby cell, which is influenced by the stimulation of the initial cell. For example, for a 15x15 2-D tissue, when cell, N = (*N*^2^ + 1)/2 = 113 is excited, the *T*_*adj*_ can be
  $$\begin{array}{c} T_{adj}=\left|T_{p_{mid}}-T_{p_{mid+1}}\right| \end{array}$$ (24)
  or
  $$\begin{array}{c} T_{adj}=\left|T_{p_{mid}}-T_{p_{mid-1}}\right| \end{array}$$ (25)
  or
  $$\begin{array}{c} T_{adj}=\left|T_{p_{mid}}-T_{p_{mid-15}}\right| \end{array}$$ (26)
  or
  $$\begin{array}{c} T_{adj}=\left|T_{p_{mid}}-T_{p_{mid+15}}\right| \end{array}$$ (27)
  where $T_{p_{mid}}$ is the peak time at cell (*N*^2^ + 1)/2 = 113, $T_{p_{mid-1}}$ is the peak time at cell ((*N*^2^ + 1)/2)-1 = 112, $T_{p_{mid-15}}$ is the peak time at cell ((*N*^2^ + 1)/2)-15 = 98 and $T_{p_{mid+15}}$ is the peak time at cell ((*N*^2^ + 1)/2) + 15 = 128 for a 15x15 2-D tissue.

For all the Eqs 24–27, *T*_*adj*_ should be the same in all the cases.

For creating the wavefront propagation of 2-D FM ventricular myocyte, we have found that the minimum value for the diffusion constant, D, equals 0.0625. We calculated the metrics for all conditions discussed using this value and the step time of 0.4 ms as a constant. Table 9 displays these values when stimulated by the cell (*N*^2^ + 1)/2. We can observe that the values of $T_{p_{mid}}$ and *T*_*adj*_ are the same, i.e., 7.6 ms and 4.8 ms, respectively, despite the alteration in the size of the 2-D FM tissue. This observation shows that when the cell in the middle is stimulated, the wavefront propagates despite the alteration in size. In all cases, the values of $T_{p_{1}}$ and $T_{p_{NN}}$ increase as the size of the 2-D FM tissue expands. Despite the observation of the surge, the individual values of $T_{p_{1}}$ and $T_{p_{NN}}$ are similar for a 7x7,15x15 and 31x31 2-D FM tissue depicting that the wavefront propagates outwardly. These properties are also observed when we tabulate the values for *T*_*mc*1_ and *T*_*mNN*_. As the size of the tissue expands from 7x7 to 15x15, the values of $T_{p_{1}}$, $T_{p_{NN}}$, *T*_*mc*1_ and *T*_*mNN*_ is found to be doubled. The parameter further doubles as the size expands from 15x15 to 31x31. This analysis shows that the 2-D diffusion equation works for the FM ventricular tissue. It also displays the variations of the parameters when cell 1 is stimulated for a diffusion constant of 0.0625 and a step time of 0.4 ms. In this case, the parameter $T_{p_{1}}$ is observed to be the same in all cases. Hence, we can infer that we have stimulated cell 1 in all the cases. The values of *T*_*adj*_ are similar despite the alterations in the size of the 2-D FM tissue. In all cases, the values of $T_{p_{mid}}$ are lesser than that of $T_{p_{NN}}$, showing proof of how the wavefront propagates when cell 1 is stimulated. The values of *T*_*mc*1_ and *T*_*mNN*_ doubles when the size of the 2-D FM tissue evolves from 7x7 to 15x15. These values tend almost to quintuple as the 2-D FM tissues expand from 7x7 to 31x31. Similar observation can be inferred for $T_{p_{mid}}$ and $T_{p_{NN}}$. Through this analysis, we can quantitatively comprehend the evolution of the wavefront propagation when cell 1 is stimulated. It also shows the variation of the same metrics when the cell *N*^2^ is stimulated using the same diffusion constant and step time. In this case, the value of $T_{p_{NN}}$ is the same, showing that the cell *N*^2^ is stimulated. The value of $T_{p_{1}}$ in this case is found to be equal to that of the $T_{p_{NN}}$ when cell 1 is stimulated. *T*_*pmid*_ is the same as the values found when cell 1 is stimulated. *T*_*mc*1_ is similar to that of *T*_*mNN*_ when cell 1 is excited. Moreover, *T*_*mNN*_ is equal to *T*_*mc*1_ when cell 1 is excited. This tabulation infers that the wavefront propagation when cell *N*^2^ is stimulated is opposite to the wavefront propagation when cell 1 is stimulated. When cells 1 to N are stimulated in the vertical direction, we get the parameters shown in Table 9. In this case, the values of $T_{p_{1}}$ and *T*_*adj*_ are the same across all the 2-D model sizes. *T*_*mc*1_ and *T*_*mNN*_ are the same despite the alteration in the size of the 2-D tissue. The $T_{p_{mid}}$ and $T_{p_{NN}}$ doubles as the tissue size alters from 7x7 to 15x15. The values almost double when the 2-D FM tissue size alters from 15x15 to 31x31. This analysis explains how the values of the respective parameters alter when a set of cells from 1 to N is stimulated.

**Table 9:** The parameters of time for 7x7,15x15 and 31x31 2-D O’Hara tissue when the diffusion constant, D = 0.0625 for different junctures of stimulation. The step time is 0.4 ms.

Stimulation	2-D model size	$T_{p_{1}}$ (ms)	$T_{p_{mid}}$ (ms)	$T_{p_{NN}}\left(ms\right)$	*T*_*mc*1_ (ms)	*T*_*mNN*_ (ms)	*T*_*adj*_ (ms)
Cell (*N*^2^ + 1)/2	7x7 2-D tissue	26.0000	7.6000	26.0000	18.4000	18.4000	4.8000
	15x15 2-D tissue	48.4000	7.6000	48.4000	40.8000	40.8000	4.8000
	31x31 2-D tissue	93.2000	7.6000	93.2000	85.6000	85.6000	4.8000
Cell 1	7x7 2-D tissue	7.6000	26.0000	42.8000	18.4000	16.8000	4.8000
	15x15 2-D tissue	7.6000	48.4000	87.6000	40.8000	39.2000	4.8000
	31x31 2-D tissue	7.6000	93.2000	177.2000	85.6000	84.0000	4.8000
Cell *N*^2^	7x7 2-D tissue	42.8000	26.0000	7.6000	16.8000	18.4000	4.8000
	15x15 2-D tissue	87.6000	48.4000	7.6000	39.2000	40.8000	4.8000
	31x31 2-D tissue	177.2000	93.2000	7.6000	84.0000	85.6000	4.8000
Cell 1 to N	7x7 2-D tissue	7.6000	22.0000	36.4000	14.4000	14.4000	4.8000
	15x15 2-D tissue	7.6000	41.2000	74.8000	33.6000	33.6000	4.8000
	31x31 2-D tissue	7.6000	79.6000	151.6000	72.0000	72.0000	4.8000

Through these tabulations, we found that *T*_*adj*_ is the same despite the alterations in size and the dimension of the tissue when we subject it to D = 0.0625 and step time = 0.4 ms. Table 10 shows that no *T*_*adj*_ exists when we make the diffusion constant D lesser than 0.0625. Hence, we assign 0.0625 as the minimum diffusion constant, *D*_*min*_. At *D*_*min*_, we already found that *T*_*adj*_ was equal to 4.8 ms. When *D*_*min*_ is doubled, we can infer that *T*_*adj*_ reduces by 2.8 ms. The *T*_*adj*_ at *D*_*min*_ is triple the values of *T*_*adj*_ found when diffusion constant is 3x*D*_*min*_ and 4x*D*_*min*_. After 5x*D*_*min*_, the value of *T*_*adj*_ is constant at 1.2 ms because the slope of the upstroke dominates the dynamics.

**Table 10:** The variation of the relative adjacent peak time, *T*_*adj*_ when the diffusion constant, D is varied according to *D*_*min*_ = 0.0625. The step time is 0.4 ms.

Diffusion constant,D	*T*_*adj*_ (ms)
< *D*_*min*_	-
*D*_*min*_	4.8000
2x*D*_*min*_	2.0000
3x*D*_*min*_	1.6000
4x*D*_*min*_	1.6000
5x*D*_*min*_	1.2000
6x*D*_*min*_	1.2000
7x*D*_*min*_	1.2000
8x*D*_*min*_	1.2000
9x*D*_*min*_	1.2000
10x*D*_*min*_	1.2000

**Fig 18:**
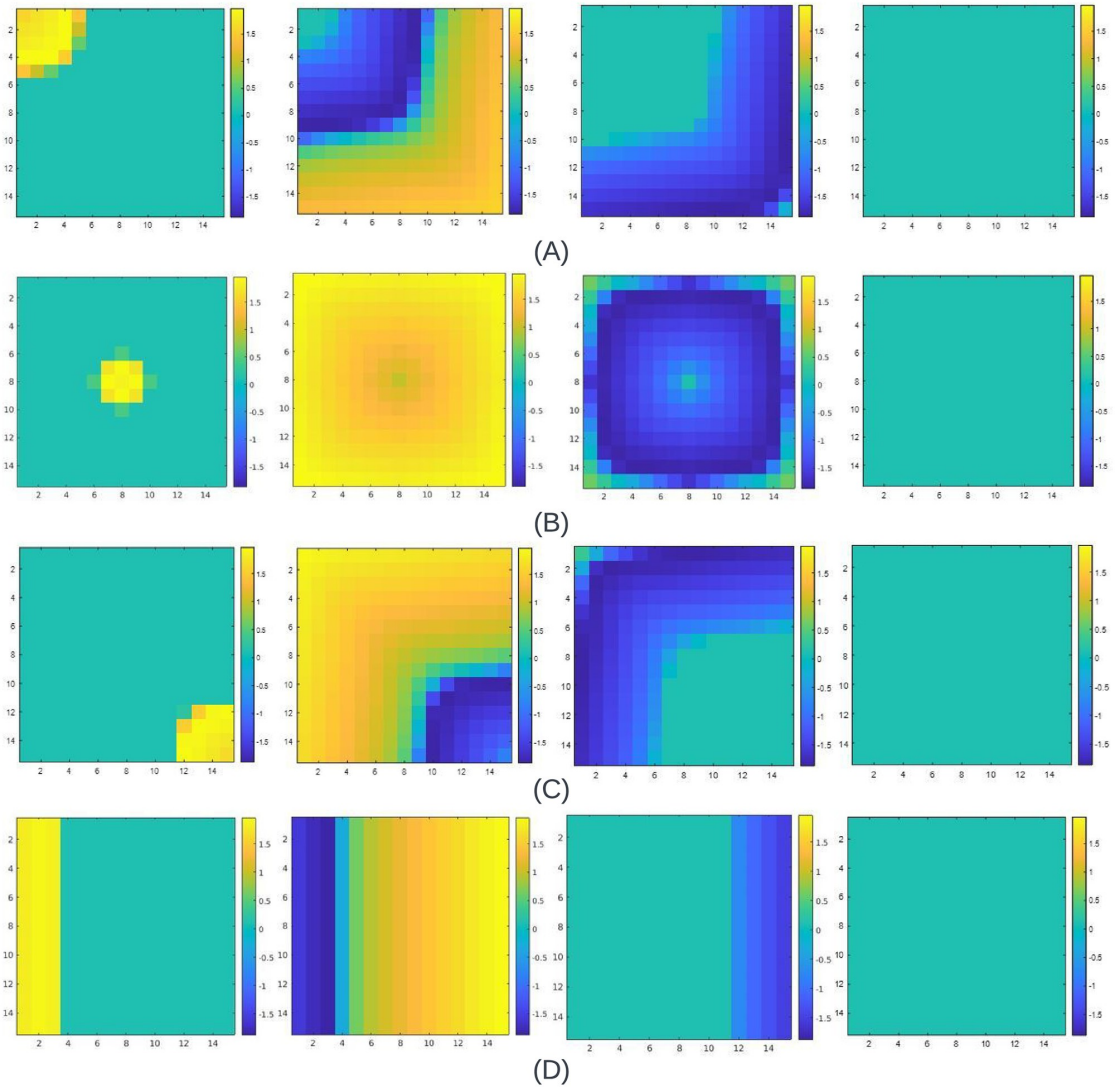
The propagation wavefront of a 15x15 2-D FM tissue of the FHN model when the (A) Cell 1 is stimulated (B) Cell (*N*^2^ + 1)/2 is stimulated, i.e., Cell 113 for a 15x15 cell matrix is stimulated (C) Cell NxN, i.e., 225 is stimulated and (D) Multiple cells from Cells 1 to N are stimulated, and N = 15 is stimulated. The propagation wavefront starts differently for each stimulation, but in the end, all the cells in the 2-D when the cell or cells are stimulated to go back to rest, tissue goes to rest. The diffusion constant, D used in all cases is 0.00375, and the step time is 0.1 ms.

Fig 18 showcases the propagation wavefront across 4 major junctures, i.e., at cell 1, cell (*N*^2^ + 1)/2, i.e., 113, cell *N*^2^ = 225 and from cells 1 to N in the vertical direction where N = 15. Fig 18(A) illustrates the wavefront propagation of a 15x15 2-D FM tissue when cell 1 is stimulated. We can observe that the wavefront propagates diagonally and gradually stimulates the adjacent cells. This propagation occurs till cell *N*^2^ = 225. As the APD state of each cell is completed, we can see that the cells within the 2-D FM tissue gradually come to rest. Afterward, we stimulated the cell (*N*^2^ + 1)/2, and the wavefront propagation is displayed in Fig 18(B). The wavefront is observed to propagate outwardly, stimulating all the cells adjacent to the cell stimulated initially. As the APD phase of all the cells is completed in sequence, the cells in the 2-D tissue gradually come to rest. When the cell *N*^2^, i.e., 225, is stimulated, we acquire the wavefront propagation shown in Fig 18(C). We can see that the wavefront propagation is the antithesis of the wavefront propagation found in Fig 18(A). The wavefront propagates diagonally, stimulating the surrounding cells from cell 225 to cell 1. After completing the APD state, all the tissue cells gradually go to the resting state. As cells 1 to 15 are stimulated in the vertical direction, the wavefront propagation is found to be as illustrated in Fig 18(D). The wavefront linearly propagates, stimulating all the cells within the 2-D FM tissue. Afterward, we can observe that all the cells gradually move to the resting state. Similar inferences were found for a 7x7 and 31x31 2-D FM tissue.

To create the wavefront propagation of 2-D FM tissue for the FHN model, we utilized the diffusion constant D as 0.00375. We calculated the metrics for all conditions using this value for a step time of 0.1 ms. Table 11 showcases all the parameters above when the cell (*N*^2^ + 1)/2 is stimulated. The *T*_*adj*_ values remain the same through the tabulation. $T_{p_{mid}}$ is the same despite the alterations in the size of the 2-D tissue, conveying that the cell (*N*^2^ + 1)/2 is stimulated in all the tissue models. In all 2-D model sizes, we can observe that $T_{p_{1}}$ and $T_{p_{NN}}$ are found to be the same. Moreover, *T*_*mc*1_ and *T*_*mNN*_ are also found to be similar, showcasing the outward propagation of the wavefront when the cell (*N*^2^ + 1)/2 is stimulated. Table 11 also showcases all the metrics when cell 1 is stimulated for all the 2-D model sizes. In this case, we can see that $T_{p_{1}}$ is the same despite the alteration in the 2-D model size. The *T*_*adj*_ is similar in all attempted conditions. $T_{p_{mid}}$, $T_{p_{NN}}$, *T*_*mc*1_ and *T*_*mNN*_ is found to double when the size of the 2-D tissue alters from 7x7 to 15x15. These almost double when the size alters from 15x15 to 31x31. When cell *N*^2^ is stimulated, we acquire the metric as tabulated in Table 11. In this case, $T_{p_{NN}}$ is the same across all the model sizes discussed. This observation shows that the wavefront propagates when cell *N*^2^ is stimulated. Interestingly, the values of *T*_*p*1_ are equal to the $T_{p_{NN}}$ and vice-versa when cell 1 is excited. All the values of $T_{p_{mid}}$ are similar to the ones found when cell 1 is stimulated. *T*_*mc*1_ is found to the same as that of *T*_*mNN*_ as cell 1 is excited. Similar observations can be found when we compare the *T*_*mNN*_ values to *T*_*mc*1_ to the cell 1 stimulation. Overall, this tabulation proves that the wavefront propagation when cell *N*^2^ is the antithesis of the wavefront propagation when cell 1 is stimulated. Moreover, it also showcases the metrics when a set of cells from 1 to N is stimulated vertically across different 2-D model sizes. We can see that the values of *T*_*adj*_ and $T_{p_{1}}$ are the same across all the 2-D tissues discussed. We also observed that *T*_*mc*1_ and *T*_*mNN*_ are the same in 7x7,15x15 and 31x31 2-D tissue. $T_{p_{mid}}$ and $T_{p_{NN}}$ tend to double when the 2-D tissue size alters from 7x7 to 15x15. These tend to double further when the tissue size is enlarged to 31x31.

**Table 11:** The parameters of time for 7x7,15x15 and 31x31 2-D FHN tissue when the diffusion constant, D = 0.00375 for stimulations at various junctures. The step time is 0.1 ms.

Stimulation	2-D model size	$T_{p_{1}}$ (ms)	$T_{p_{mid}}$ (ms)	$T_{p_{NN}}\left(ms\right)$	*T*_*mc*1_ (ms)	*T*_*mNN*_ (ms)	*T*_*adj*_ (ms)
Cell (*N*^2^ + 1)/2	7x7 2-D tissue	10.4000	2.5000	10.4000	7.9000	7.9000	2.0000
	15x15 2-D tissue	19.6000	2.5000	19.6000	17.1000	17.1000	2.0000
	31x31 2-D tissue	38.0000	2.5000	38.0000	35.5000	35.5000	2.0000
Cell 1	7x7 2-D tissue	2.5000	10.4000	17.3000	7.9000	6.9000	2.0000
	15x15 2-D tissue	2.5000	19.6000	35.7000	17.1000	16.1000	2.0000
	31x31 2-D tissue	2.5000	38.0000	72.5000	35.5000	34.5000	2.0000
Cell *N*^2^	7x7 2-D tissue	17.3000	10.4000	2.5000	6.9000	7.9000	2.0000
	15x15 2-D tissue	35.7000	19.6000	2.5000	16.1000	17.1000	2.0000
	31x31 2-D tissue	72.5000	38.0000	2.5000	35.5000	34.5000	2.0000
Cells 1 to N	7x7 2-D tissue	2.5000	8.5000	14.5000	6.0000	6.0000	2.0000
	15x15 2-D tissue	2.5000	16.5000	30.5000	14.0000	14.0000	2.0000
	31x31 2-D tissue	2.5000	32.5000	62.5000	30.0000	30.0000	2.0000

Throughout all these analyses, we utilized the diffusion constant, D = 0.00375, and we found *T*_*adj*_ to be the same for the various conditions explained in the tabulations. On reducing this value further, we found that the cell-to-cell propagation does not occur when D < 0.003673. Hence for Table 12, we took the minimum diffusion constant, *D*_*min*_ as 0.003673. We found that *T*_*adj*_ was 0.5 ms higher than that of the *T*_*adj*_ found when D = 0.00375. As the value of D is twice that of *D*_*min*_, we can see that *T*_*adj*_ gets reduced by 1.5 ms. *T*_*adj*_ reduces further by 0.2 ms when D is thrice that of *D*_*min*_. At 4x*D*_*min*_ and 5x*D*_*min*_, the *T*_*adj*_ remains the same, i.e., 0.6 ms. *T*_*adj*_ reduces by 0.1 ms when D = 6x*D*_*min*_, D = 7x*D*_*min*_ and D = 8x*D*_*min*_. At D = 9x*D*_*min*_ and D = 10x*D*_*min*_, *T*_*adj*_ further reduces by another 0.1 ms. This tabulation infers that *T*_*adj*_ is inversely proportional to D.

**Table 12:** The variation of the relative adjacent peak time, *T*_*adj*_ when the diffusion constant, D is varied according to *D*_*min*_ = 0.003673. The step time is 0.1 ms.

Diffusion constant,D	*T*_*adj*_ (ms)
< *D*_*min*_	-
*D*_*min*_	2.5000
2x*D*_*min*_	1.0000
3x*D*_*min*_	0.8000
4x*D*_*min*_	0.6000
5x*D*_*min*_	0.6000
6x*D*_*min*_	0.5000
7x*D*_*min*_	0.5000
8x*D*_*min*_	0.5000
9x*D*_*min*_	0.4000
10x*D*_*min*_	0.4000

#### Effects of cellular dysfunctions

Previously, we discussed various parameters in terms of time that can be used to justify a 2-D FM tissue. This part will explore how these parameters vary when certain cellular dysfunctions are introduced to the 2-D tissue. A 17x17 2-D FM tissue is created using O’Hara [7] and FHN cell models. The 17x17 2-D FM tissues are arranged in a systematic pattern. The top-left corner of the 2-D tissue is cell 1. At the same time, the bottom-right corner of the tissue is a cell *N*^2^. The middle cell uses (*N*^2^ + 1)/2, i.e., N = 145.

**Fig 19:**
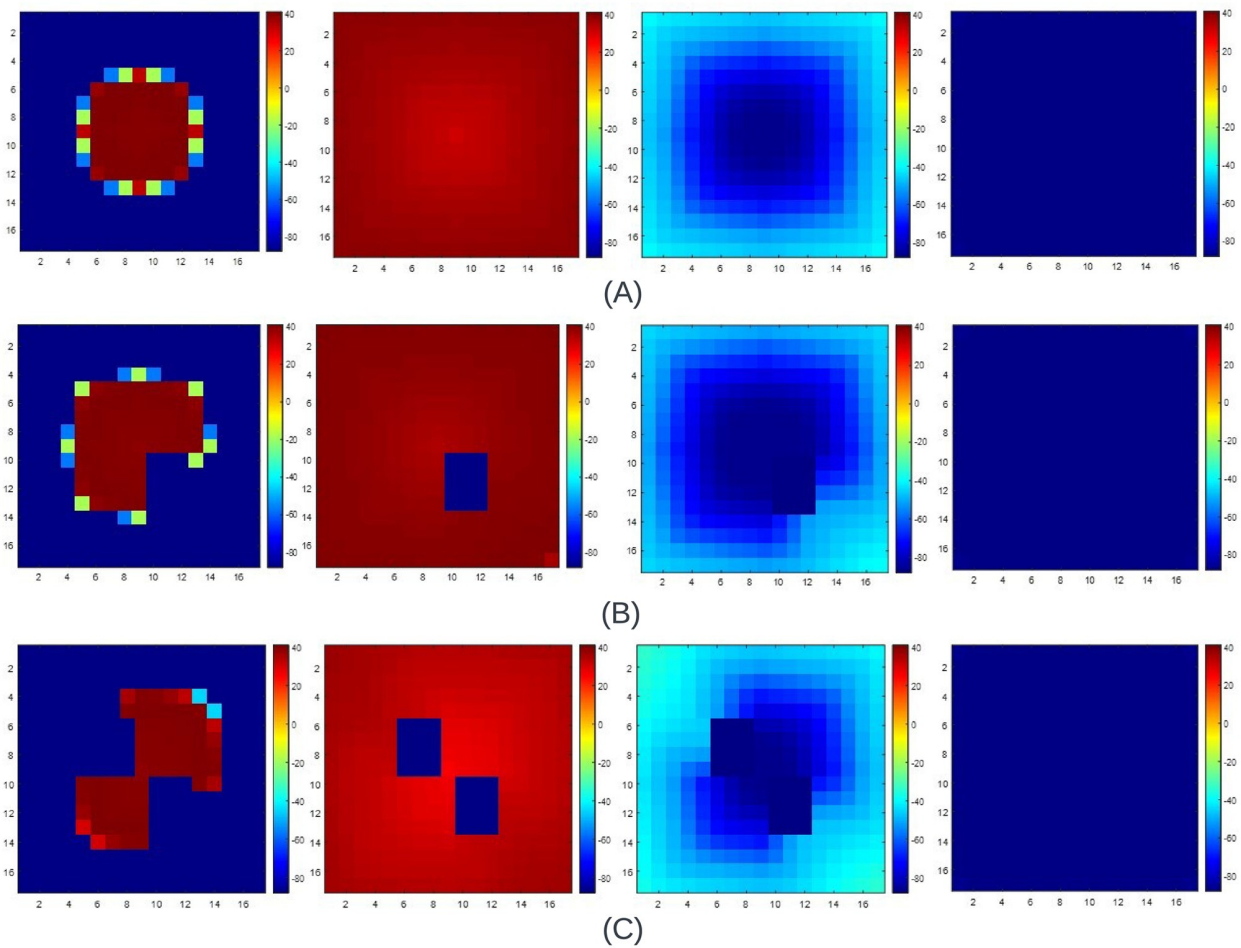
The propagation wavefront of a 17x17 2-D FM tissue when the cell, (*N*^2^ + 1)/2 = 145, is stimulated when there are (A) no dysfunctions, (B) One dysfunction, (C) Two dysfunction. The diffusion constant, D, used in all cases is 0.0625, and the step time is 0.4 ms.

Fig 19 (refer movies in S1, S3 and S4 Movies) illustrates the wavefront propagation for a 17x17 2-D with and without cellular dysfunctions. The stimulus is exerted on the cell 145 to understand the variations in the wavefront propagation with and without cellular dysfunction. Fig 19(A) shows the wavefront propagation of a 17x17 2-D FM tissue when there is no cellular dysfunction. The wavefront evolves outwardly, as observed in the previous sections. In Fig 19(B), we induce specific cellular dysfunctions in cells beneath the cell 145. In this case, the wavefront does not propagate through these cells. The wavefront propagates to the other functional cells but bypasses the cells with dysfunctions. As cell 145 goes to rest, all the tissue within the 2-D FM tissue goes to the resting state. We introduce two sets of cellular dysfunctions, and the wavefront propagation is illustrated in Fig 19(C). In this case, the wavefront bypasses the dysfunctional set of cells above and below the cell 145. The wavefront evolves as usual in the other cells within the 2-D FM tissue. As each cell within the 2-D FM tissue goes to the resting state, the entire set of cells, barring those with dysfunctions, are found to go towards the resting state gradually.

The tabulations of these illustrations are depicted in Table 13. The wavefront without the cellular dysfunction is observed to have the same value for $T_{p_{1}}$ and $T_{p_{NN}}$ which in turn leads to the values of *T*_*mc*1_ and *T*_*mNN*_ to be equal. However, when the dysfunctions in these cells are introduced, as per Fig 19(B), we can observe that there is a surge in the values of $T_{p_{NN}}$ and *T*_*mNN*_ by 6.4 ms. When two sets of cellular dysfunctions exist, as in Fig 19(B), we can see the overall increase in the values of $T_{p_{1}}$ and $T_{p_{NN}}$, which in turn leads to the surge in the values of *T*_*mc*1_ and *T*_*mNN*_. However, the values of $T_{p_{mid}}$ and *T*_*adj*_ remain the same in all the cases. Overall, there is some drastic alteration in the mentioned parameters when certain cellular dysfunctions are introduced. We then look to comprehend the wavefront propagation using the FHN model in a 2-D FM tissue where similar cellular dysfunction can be done.

**Table 13:** The time parameters for 17x17 2-D FM tissue using different cell models with diffusion constant, D and step time when the middle cell (*N*^2^ + 1)/2 = 145 is stimulated with and without cellular dysfunctions.

Cell model	D	Step Time (ms)	Dysfunction	$T_{p_{1}}$ (ms)	$T_{p_{mid}}$ (ms)	$T_{p_{NN}}\left(ms\right)$	*T*_*mc*1_ (ms)	*T*_*mNN*_ (ms)	*T*_*adj*_ (ms)
O’Hara [7]	0.0625	0.4	None	54.0000	7.6000	54.0000	46.4000	46.4000	4.8000
			One set	54.0000	7.6000	60.4000	46.4000	52.8000	4.8000
			Two sets	62.0000	7.6000	60.4000	54.4000	52.8000	4.8000
FHN	0.003673	0.1	None	25.6000	2.5000	25.6000	23.1000	23.1000	2.5000
			One set	25.6000	2.5000	29.4000	23.1000	26.9000	2.5000
			Two sets	30.3000	2.5000	29.4000	27.8000	26.9000	2.5000

**Fig 20:**
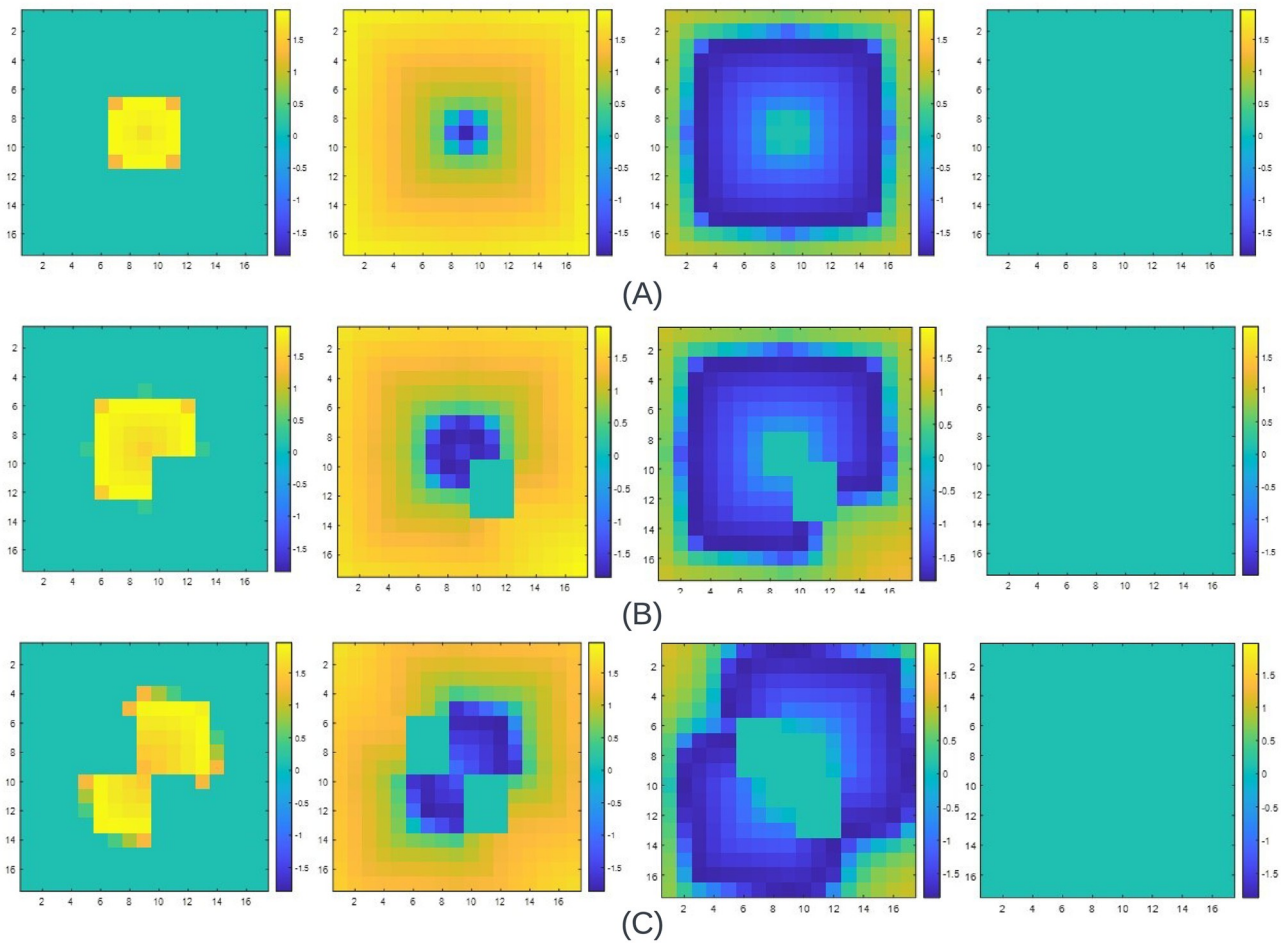
The propagation wavefront of a 17x17 2-D FM tissue using FHN model when the cell (*N*^2^ + 1)/2 = 145 is stimulated when there are (A) no dysfunctions (B) One dysfunction (C) Two dysfunction. The diffusion constant, D, used in all cases is 0.003673, and the step time is 0.1 ms.

Fig 20 (refer movies in S2, S5 and S6 Movies) depicts the wavefront propagation of the FHN model using a 17x17 2-D FM tissue. As mentioned earlier, the minimum diffusion constant, *D*_*min*_, was found to be 0.003673. Hence, we will utilize this value for a time step of 0.1 ms. The wavefront evolves outwardly in all directions when no cellular dysfunction exists, as depicted in Fig 20(A). When cellular dysfunction exists, as in Fig 20(B), we can observe that the wavefront propagation tends to elude these cells. The other functional cells tend to propagate outwardly, and all of them are found to move gradually toward the resting state when there are two cellular dysfunctions, as shown in Fig 20(C), the wavefront propagates by eluding these areas. As another cell, 145, goes to rest, all the functional cells surrounding it go to the resting state. From this illustration, we can comprehend that the wavefront propagation evolves differently whenever a set of cells is dysfunctional.

Without introducing certain dysfunctions to the 2-D FM model using the FHN AP characteristics, we can observe that the parameters *T*_*adj*_ and *T*_*pmid*_ remain the same (refer Table 13). The values of $T_{p_{1}}$ and $T_{p_{NN}}$ are the same, which in turn leads to the parameters *T*_*mc*1_ and *T*_*mNN*_ being similar. We can observe that the parameter $T_{p_{NN}}$ tends to surge by 3.8 ms, leading to the alteration in the value of *T*_*mNN*_ by the same margin as dysfunctions are introduced as in Fig 20(B). In this case, the value of $T_{p_{1}}$ and $T_{p_{mid}}$ is the same as the wavefront propagation in Fig 20(A). As cellular dysfunctions are introduced in Fig 20(C), we can observe that the values of $T_{p_{1}}$ and $T_{p_{NN}}$ tend to rise, which leads to the rise in the values of *T*_*mc*1_ and *T*_*mNN*_. We can see that certain parameters tend to increase when cellular dysfunctions occur in the 2-D FM tissues using the FHN cell model.

Further investigation into the S1-S2 protocol and APD restitution [65] is required to calculate all the time parameters like the other 2-D tissue models. Overall, cellular dysfunctions affect the wavefront propagation in a 2-D for different cells.

## Discussion

This study presents a novel FM model designed to alter the characteristics of a single sine wave based on the APDs of a cell model: Beeler Reuter (BR), Fenton Karma (FK), FitzHugh Nagumo and Fabbri et al. [6] model. Before evaluating how precisely this novel methodology can replicate the APD, various test signals were used to comprehend the efficacy of this method. Tables 2 and 3 tabulates various values, which showcases how this model effectively replicates the test signals.

Afterward, we reconstruct the Δ_*w*_ and APD waveshapes for the four cell models to further confirm the model’s effectiveness. The RMSE metrics in Table 6 are absolute measurements. The percentage error for the Δ_*w*_ and the APD for the BR model are 1.28% and 3.6%, respectively. The closeness of the traces in Figs 8 and 9 reflect these percentage errors. The high *R*^2^ metrics for Δ_*w*_ and the APD reconstructions confirm the fidelity of the reconstructed signals using the FM methodology. Notably, the FM model can capture any dynamic changes in the morphologies by adapting the frequency modulating factor, Δ_*w*_. Hence, this model can potentially simulate the effects of new drugs with the *I*_*f*_ blockade, $I_{f_{b}}$ (refer Table 5).

Utilizing a piecewise linear segment reduces the Look-Up-Table (LUT) size from K samples to n segments. For example, the Δ_*w*_ profile has 30000 samples for the BR model and would require an LUT with 30000 elements. Using piecewise linear fitting, the LUT size equals the number of segments (refer Table 6).

We introduced a state controller to the FM model to facilitate the interconnection of cells and, hence, implement various tissue models. Figures showing wavefront propagation for 1-D and 2-D tissues have been presented.

## Conclusion

The proposed FM model needs to be reported in the literature. It accurately captures the electrical activities of various types of biological cells. It replicates the action potential durations (APDs) and precisely accounts for the effects in the blockage current. The performance of this model closely aligns with biophysical simulations and clinical data.

The model is inherently simple and holds significant potential for emulating multiple cells, thereby performing tissue-level simulations. Implementing this model is straightforward and enables a seamless application to various fields where processes exhibit (non-linear) periodic or quasi-periodic characteristics. This adaptability can facilitate advancement in various research domains and enhance real-time emulations, especially on an FPGA.

## Future scope

This study shows the successful simulation of the proposed modeling approach to replicate a cell model’s AP precisely. Although dynamic properties, like APD restitution and action potential rate dependence, have not been included in this study, the state controller can be enhanced (as has been done for $I_{f_{b}}$) to incorporate these properties. Given the generic nature of this model, it can be expanded to various biological cells like skeletal muscle cells, atrial myocytes, plant cells, and even the cells of diverse species.

The proposed novel FM model has the potency for a low-cost computational approach that may allow researchers to explore new avenues like emulation of these on an FPGA. This area requires an in-depth investigation to guarantee maximum effectiveness and efficiency. Moreover, the fitting piecewise polynomial can be altered from linear to quadratic or cubic to reduce the number of breakpoints, which may provide a more accurate APD waveshape.

## Supporting information

S1 Movie Propagation wavefront of a 17x17 2-D tissue of the O’Hara [7] model when the stimulation is done on cell, N = (*N*^2^ + 1)/2 where diffusion constant, D = 0.0625 and step time is 0.4 ms. Movie illustrates the propagation wavefront of a 17x17 2-D tissue of the O’Hara [7] model when cell N = (*N*^2^ + 1)/2 is stimulated.
(AVI)

S2 Movie Propagation wavefront of a 17x17 2-D tissue of the FHN model when the stimulation is done on cell, N = (*N*^2^ + 1)/2. Diffusion constant, D is 0.003673 and step time is 0.1 ms. Movie illustrates the propagation wavefront of a 15x15 2-D tissue of the FHN model when cell N = (*N*^2^ + 1)/2 is stimulated.
(AVI)

S3 Movie The propagation wavefront of a 17x17 2-D FM tissue using O’Hara [7] model when the cell, N = (*N*^2^ + 1)/2 = 145 is stimulated when there is one set of cellular dysfunction. Diffusion constant, D is 0.0625 and step time is 0.4 ms. Movie shows how the propagation wavefront for a 17x17 2-D tissue alters when there is a set of cellular dysfunctions.
(AVI)

S4 Movie The propagation wavefront of a 17x17 2-D FM tissue using O’Hara [7] when the cell, N = (*N*^2^ + 1)/2 = 145 is stimulated when there are two sets of cellular dysfunction. Diffusion constant, D is 0.0625 and step time is 0.4 ms. Movie shows how the propagation wavefront for a 17x17 2-D tissue alters when there are two sets of cellular dysfunctions.
(AVI)

S5 Movie The propagation wavefront of a 17x17 2-D FM tissue using FHN model when the cell, N = (*N*^2^ + 1)/2 = 145 is stimulated when there is one set of cellular dysfunction. Diffusion constant, D is 0.003673 and step time is 0.1 ms. Movie shows how the propagation wavefront for a 17x17 2-D tissue alters when there is a set of cellular dysfunctions.
(AVI)

S6 Movie The propagation wavefront of a 17x17 2-D FM tissue using FHN model when the cell, N = (*N*^2^ + 1)/2 = 145 is stimulated when there are two sets of cellular dysfunction. Diffusion constant, D is 0.003673 and step time is 0.1 ms. Diffusion constant, D is 0.003673 and step time is 0.1 ms. Movie shows how the propagation wavefront for a 17x17 2-D tissue alters when there are two sets of cellular dysfunctions.
(AVI)
